# A Combined LC-MS Metabolomics- and 16S rRNA Sequencing Platform to Assess Interactions between Herbal Medicinal Products and Human Gut Bacteria *in Vitro*: a Pilot Study on Willow Bark Extract

**DOI:** 10.3389/fphar.2017.00893

**Published:** 2017-12-13

**Authors:** Eva-Maria Pferschy-Wenzig, Kaisa Koskinen, Christine Moissl-Eichinger, Rudolf Bauer

**Affiliations:** ^1^Department of Pharmacognosy, Institute of Pharmaceutical Sciences, Universtity of Graz, Graz, Austria; ^2^Section of Infectious Diseases and Tropical Medicine, Department of Internal Medicine, Medical University of Graz, Graz, Austria; ^3^BioTechMed-Graz, Graz, Austria

**Keywords:** LC-MS metabolomics, 16S rRNA sequencing, human gut bacteria, willow bark extract, herbal medicinal product, gut microbiome

## Abstract

Herbal preparations are complex mixtures of natural products, many of which are able to reach the distal gut due to low oral bioavailability. There, they can influence the microbial communities, and can be metabolized into potentially absorbable bioactive compounds by the intestinal bacteria. This aspect has often been disregarded when searching for the active principles of medicinal plants and herbal medicinal products. The aim of this study was to establish an interdisciplinary platform to unravel interactions of herbal medicine and intestinal microbiota, using a combined LC-MS metabolomics and 16S rRNA microbiome sequencing approach. Willow bark extract (WBE), a herbal medicinal product with a long history of traditional use and a well-established anti-inflammatory activity, was incubated with human fecal suspension under anoxic conditions. Samples were taken after 0.5, 4, and 24 h of incubation. Microbiome analyses revealed that incubation with WBE had a marked effect on microbial community composition and functions. For example, the proportion of *Bacteroides* sp. was clearly enhanced when the fecal sample used in this study was incubated with WBE. LC-MS analysis showed that WBE constituents were readily metabolized by fecal bacteria. Numerous microbial metabolites could be annotated, allowing the construction of putative microbial degradation pathways for the main groups of WBE constituents. We suggest that studies of this type help to increase the knowledge on bioactive principles of medicinal plants, since gut microbial metabolites might have been underestimated as a source of bioactive compounds in the past.

## Introduction

The human body hosts at least as many microbial as human cells (Sender et al., 2016). Microorganisms reside on our skin, in mouth, nose, ears, intestinal tract and genitals. Many of these microbial species are essential for our survival, health and well-being. The human gut microbiome is known to influence the host's immune system, development and physiology, as well as metabolism (de Vos and de Vos, 2012). The composition of the microbial community in the human gut is highly variable between individuals (Arumugam et al., 2011) and is strongly influenced by factors like age (O'Toole and Jeffery, 2015), diet (Moschen et al., 2012), and health status (Shreiner et al., 2015).

Compounds ingested as food or drugs can influence the human microbial community. Either, certain microbes are inhibited (by e.g., antibiotics), or stimulated by the provision of specific carbon- and energy sources (e.g., prebiotics such as inulin). On the other hand, many compounds, in particular natural products ingested via diet or herbal medicines, reach the colon due to their low oral bioavailability and are readily metabolized by gut microbiota. In many cases, they are decomposed to metabolites with lower molecular weight and polarity, and consequently better bioavailability. Many herbal preparations have a thousands-of-years old tradition of medicinal use, and still, in many cases, their pharmacologically active principles are unknown. Currently, microbial metabolites are in the spotlight of medicinal plant research, since they might be of importance to explain the pharmacological activity of some of these traditional herbal preparations (Possemiers et al., 2011; Mena et al., 2015; Chen et al., 2016).

Composition and activity of the human gut microbial community can sometimes be a key factor in the therapeutic effectiveness of natural products. As an example, the isoflavone daidzin can be metabolized by gut microbiota either to O-desmethylangolesin, or to S-(-)-equol, the latter one currently being regarded as responsible for the endocrine-related benefits of soy consumption. Interestingly, only 25–30% of individuals in Western countries have the ability to produce S-(-)-equol, a fact that has been neglected in many earlier clinical studies on isoflavones (Shor et al., 2012; Sánchez-Calvo et al., 2013). Another example for microbial bioactivation are ellagitannins, high molecular weight polyphenolic compounds that are contained at substantial amounts in many medicinal plants and plant derived food, like berries, nuts, and oak-aged wines. These compounds have a very low bioavailability and are therefore available to colon microbiota. Recently, urolithins have been identified as low-molecular weight microbial ellagitannin degradation products. In contrast to ellagitannins, urolithins are bioavailable and showed anti-inflammatory, antioxidant and anticancer effects *in vitro* and in animal studies (Espín et al., 2013).

The majority of studies investigating the metabolization of natural products by human fecal microbiota *in vitro* (e.g., Appeldoorn et al., 2009; Tomas-Barberan et al., 2013; Tan et al., 2014), has been performed with single compounds, and focussed on their metabolization by the microbes, but did not consider the impact of the added compounds on the microbes.

The aim of the current study was to establish an interdisciplinary platform that combines microbiomics and metabolomics techniques and thereby allows analyzing both aspects: the influence of plant constituents on intestinal microbiota, as well as the impact of intestinal microbiota on the metabolic fate of plant constituents. For the initial experiments conducted in this study, we used willow bark extract (WBE). Willow bark (*Salix* sp., Salicaceae) has been used for thousands of years as an anti-inflammatory, analgesic and antipyretic agent, and the therapeutic use of standardized willow bark extracts against lower back pain is well-established by clinical trials (Shara and Stohs, 2015)^1^. Willow bark extracts are usually standardized to their content of salicylic alcohol derivatives with the main constituent salicin. However, their clinical efficacy cannot be fully explained by the presence of this group of constituents, and other compound classes like flavonoids and proanthocyanidin derivatives are thought to contribute to the activity as well (Nahrstedt et al., 2007; Shara and Stohs, 2015). Therefore, in the current study, we aimed to investigate the whole range of constituents and their putative microbial metabolites by a metabolomics approach, and to elucidate the interplay between the extract's constituents and human gut microbiota by 16S rRNA sequencing.

## Materials and methods

### Ethics and informed consent statement

The human fecal sample was taken and handled with approval by and in accordance with the Ethics Commission at the Medical University of Graz and all experiments were performed in accordance with relevant guidelines and regulations (Reference number: 27–151 ex 14/15). The Ethics Commission stated that no ethical concerns are raised by the methods applied and approved the procedures. Written informed consent was obtained from the study participant for study participation and publication in accordance with the Declaration of Helsinki. No metadata were derived and the human material was not subject of this study.

### Chemicals and reagents

Dry, powdered ethanolic willow bark extract (WBE) (extraction solvent: 70% ethanol (v/v); drug-extract ratio: 8–14:1) was kindly provided by Bionorica (Neumarkt, Germany). Acetonitrile and methanol (gradient grade) were purchased from VWR. Formic acid (LC-MS grade) was purchased from Fluka. Ingredients for phosphate buffered saline (PBS) were purchased from VWR; the buffer was bubbled with N_2_, reduced with cysteine-HCl, and autoclaved before usage. Propidium monoazide was purchased from VWR. The ethanolic roseroot extract used for identification of cinnamyl alcohol derivatives was kindly provided by Prof. Franz Bucar (University of Graz, Austria).

### Incubation experiment

WBE was dissolved with the vehicle, a mixture of absolute ethanol and PBS buffer (1:1), to a concentration of 100 mg/ml (concentration 1); this solution was diluted with the vehicle to 20 mg/ml (concentration 2). For incubation experiments, freshly passed feces provided by a healthy volunteer (female, age 39, non-smoker, normal mixed diet, no antibiotics for more than 2 years; intake of spicy food was avoided the last days before donation) was immediately transferred to an anaerobic chamber (Don Whitley A85; gas phase N_2_H_2_CO_2_) and mixed with anoxic PBS (PBS buffer: Na_2_HPO_4_ and NaH_2_PO_4_ (both 0.2 M), titrated to a pH of 7.2; NaCl 0.13 M; reduction was performed with cysteine-HCl) to retrieve a 10% human fecal suspension (HFS).

For incubation, 27 ml of HFS were mixed with 3 ml of sample; thus the final concentration of feces in PBS was 0.1 g/ml, the end concentration of WBE in the incubation mixture was 10 mg/ml (concentration 1) and 2 mg/ml (concentration 2). The final concentration of ethanol in the incubation mixture was 5% (v/v). For investigation of microbiome changes without sample addition and to investigate the influence of vehicle substance, one incubation series was performed with vehicle only (PBS buffer and ethanol 1:1; vehicle control) instead of sample, or pure PBS buffer instead of sample (PBS control). In order to rule out changes in WBE concentration over time that are not due to HFS, one incubation was performed with 3 ml WBE (concentration 1) in 27 ml PBS buffer instead of HFS (extract control). All incubations and control experiments were performed in triplicates under physiological conditions (anoxic, 37°C).

Samples for microbiome and LC-MS analysis were taken at 0.5 h (t_0_), 4 h (t_4_), and 24 h (t_24_) after sample addition. Samples for LC-MS analysis were immediately cooled and centrifuged (10 min, 13,000 rpm, 4°C). The supernatant was sterile filtered (0.45 μm filter pore size) and frozen at −80°C until analysis. For microbiome analysis, samples for conventional total DNA extraction (for identifying the bacterial community members present), as well as samples for propidium monoazide (PMA) treatment prior to DNA extraction (to assess the community composition and diversity of only living bacterial cells) were taken. Samples for DNA extraction were immediately frozen at −80°C. PMA samples were treated with 50 μM PMA (Biotium, Fremont, USA), and incubated in the dark for 10 min (slight shaking). Afterwards, the samples were placed in the PMA-Lite LED Photolysis Device (Biotium) and treated with light for 15 min before being frozen at −80°C until their analysis.

### UHPLC-HRMS analysis and data processing

Analyses were performed on an Ultimate 3000 UHPLC hyphenated with a Q Exactive™ hybrid quadrupole orbitrap mass spectrometer (Thermo Scientific) in the ESI negative mode. As a stationary phase, a Kinetex C18 column (1.7 μm, 2.1 × 10 mm, Phenomenex) protected by a SecurityGuard UltraGuard cartridge (C18; 2.1 mm Ø; Phenomenex) was used. The mobile phase consisted of water + 0.2% formic acid (A) and acetonitrile/methanol (7/3) + 0.2% formic acid (B). The gradient was set as follows: 0–2 min, 3% B in A; 3–36 min, 3–36% B in A; 36–46 min, 36–100% B in A; 46–52 min, 100 % B in A; 52–53 min, 100–3% B in A; 53–63 min, 3% B in A. Flow rate was 0.2 ml/min and column temperature was 40°C. The mass spectrometer was run in the HESI negative mode using the following parameters: probe heater temperature 400°C; capillary temperature 275°C; spray voltage 2.5 kV, sheath gas flow 40 arbitrary units; auxiliary gas flow 12 arbitrary units; resolution: 70,000 (full MS) and 17,500 (data dependent MS^2^). Prior to injection, samples were thawed and centrifuged (13,000 rpm, 10 min). Injection volume was 1 μl for concentration 1 and 3 μl for concentration 2. As a blank, 5% ethanol in PBS buffer was injected. Raw data were deposited in MetaboLights http://www.ebi.ac.uk/metabolights/; Study Nr MTBLS479).

Data processing was done with SIEVE^TM^ 2.2 (Thermo Scientific) using the Component Extraction algorithm. The resulting frame reports consisted of all features (i.e., m/z–retention time pairs) detected in the different samples and their respective peak areas. Furthermore, ratios between sample groups (t4 vs. t0 and t4 vs. t24) were calculated, and the significance of differences between the respective groups was assessed by unpaired, two-tailed Student's *t*-test. Processed data were exported to Microsoft Excel (Supplementary Tables 1–3). Features with a t24/t0 ratio > 2 (*p* < 0.05) were regarded as significantly increasing over time; features with a t24/t0 ratio < 0.5 (*p* < 0.05) were regarded as significantly decreasing over time; features with a t4/t0 ratio >2 (*p* < 0.05) plus a t4/t24 ratio >1.25 were regarded as intermediates. Lists of features possessing these properties were generated and crosschecked with the PBS control feature list (Supplementary Table 3) in order to rule out changes over time which were not caused by incubation with HFS.

### DNA extraction of microbiota samples, next generation sequencing, and sequence processing

DNA from both sample types (PMA and untreated) was extracted using the EZNA stool DNA kit (Omega bio-tek), following the extraction protocol as given by the manufacturer. DNA concentration was determined via Qubit and a standardized amount was subjected to PCR. The 16S rRNA gene amplicons for the universal approach were amplified using Illumina-tagged primers F515 (5′-TCGTCGGCAGCGTCAGATGTGTATAAGAGACAG-GTGCCAGCMGCCGCGGTAA-3′) and R806 (5′-GTCTCGTGGGCTCGGAGATGTGTATAAGAGACAG-GGACTACHVGGGTWTCTAAT-3′) (Caporaso et al., 2012). The cycling conditions for the universal approach were: initial denaturation at 94°C for 3 min, followed by 35 cycles of denaturing at 94°C for 45 s, annealing at 60°C for 60 s and elongation at 72°C for 90 s and a final elongation step at 72°C for 10 min. Library preparation and the sequencing were carried out at the Core Facility Molecular Biology at the Center for Medical Research at the Medical University of Graz, Austria. In brief, DNA concentrations were normalized using a SequalPrep™ normalization plate (Invitrogen), and each sample was indexed with a unique barcode sequence (8 cycles index PCR). After pooling of the indexed samples, a gel cut was carried out to purify the products of the index PCR. Sequencing was done using the Illumina MiSeq device and MS-102-3003 MiSeq® Reagent Kit v3-600cycles (2 × 251 cycles).

To analyse the microbial community composition and taxonomic diversity obtained raw reads were processed through an in-house Galaxy (Hillman-Jackson et al., 2012) pipeline, using QIIME (Kuczynski et al., 2012), following the proposed Standard Operation Procedure (SOP). Sequences were clustered into OTUs at a threshold of 97%. Taxonomic assignment was performed by querying the sequence reads against GreenGenes 13_8 (McDonald et al., 2012). A biome table was constructed for downstream analyses, and OTUs represented by 5 or less sequences were removed. These data processing steps were performed in Galaxy, an open source web-based platform for data processing and analysis (Hillman-Jackson et al., 2012). This platform was made available by the Center for Medical Research (ZMF), Medical University of Graz. To calculate alpha and beta diversities, differences in community composition, and visualize the results, we applied Calypso (Version 5.8), an online platform for mining, visualizing and comparing multiple microbial community composition data (cgenome.net/calypso; Zakrzewski et al., 2017). Total-sum normalization was applied for 16S rRNA gene data.

To analyse the predicted functions of studied microbial communities, the sequence data was processed with QIIME (Caporaso et al., 2010) and closed-reference OTU picking based on GreenGenes taxonomy (13_8 database, McDonald et al., 2012). A subsequent PICRUSt (version 1.0.0.) analysis was performed using the default settings (Langille et al., 2013). Statistical comparisons were performed using LEfSe (Segata et al., 2011).

Text formatted biome tables of MiSeq sequence data have been deposited at Figshare (https://figshare.com) as dataset “BiomTable_MiSeq_Pferschy-Wenzig_et_al_2017.xlsx.” Sequence data are deposited in The European Nucleotide Archive (ENA; BioProject No.: PRJEB21169).

### Availability of data and materials

Sequence data have been deposited in The European Nucleotide Archive (ENA; BioProject No.: PRJEB21169). Text formatted biom tables of MiSeq sequence data have been deposited at Figshare (https://figshare.com) as dataset “BiomTable_MiSeq_Pferschy-Wenzig_et_al_2017.xlsx” (https://figshare.com/). A full table of all microbial taxa that were significantly changed by WBE addition are included as Supplementary Table 4. UHPLC-DAD-HRMS raw data are publicly available in MetaboLights http://www.ebi.ac.uk/metabolights/ Study Nr MTBS479. Text- formatted tables of UHPLC-DAD-HRMS data processed with SIEVE 2.2 are included in this published article as Supplementary Tables 1–3. Details on identification of WBE constituents are contained in in Supplementary Data Sheet 1.

## Results

### Addition of WBE has an effect on the composition of fecal microbial communities *in vitro*

In order to assess the potential impact of WBE on fecal microbiota, we analyzed the microbial composition of human fecal suspension (HFS) incubated with WBE at concentrations of 0 mg/ ml (PBS and vehicle control), 2 mg/ ml and 10 mg/ ml, at 0.5, 4, and 24 h. One aliquot of each sample was treated with PMA before DNA extraction, in order to mask background DNA and to sharpen the results (Nocker et al., 2007), as nearly one-third of the human gut microbial cells can be considered severely damaged (Maurice et al., 2017).

All assays were performed in triplicates, resulting in 72 individual datasets. After raw read processing, chimera checking and removal of rare reads (operational taxonomic units, OTUs, represented by 5 or less sequence reads in all samples), we analyzed more than 14,000 OTUs with according relative quantitative abundance information.

**Figure 1:**
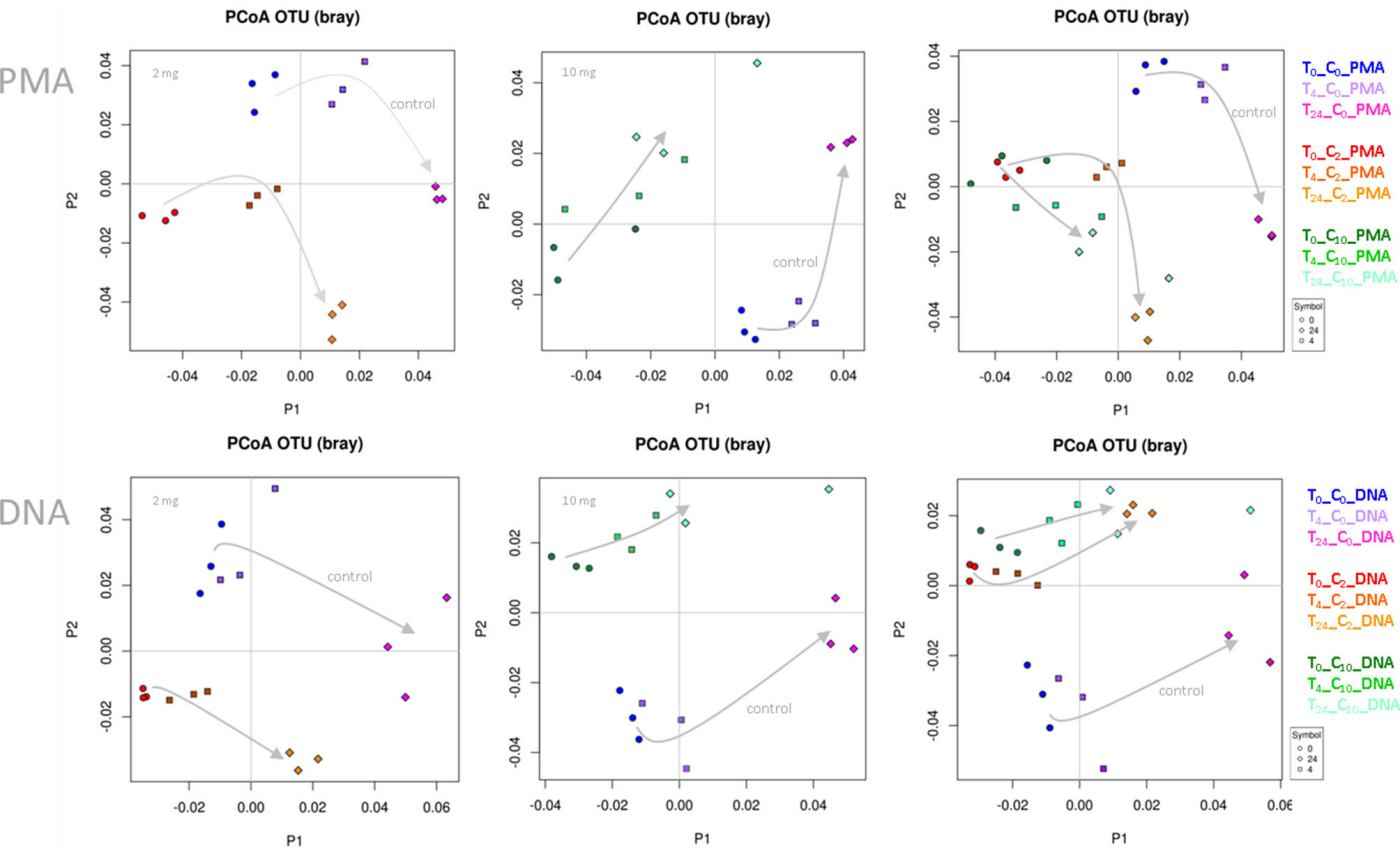
PCoA plots based on Bray-Curtis distance. Data points of WBE-treated samples clustered together and separate from control samples. The two concentrations of WBE (given in each upper left corner of the plot) affected the microbial composition differently. (C_0_, PBS control sample; C_2_, 2 mg WBE/ml HFS; C_10_, 10 mg WBE/ml HFS; DNA, PMA-untreated; PMA, PMA-treated prior to DNA extraction).

In the first step, we performed principal coordinate analyses (PCoA plots, Figure 1), in order to display the beta diversity distances of the samples. Depending on the addition of WBE or not, and on the WBE concentration, samples of the same type were clearly clustering together (and separate from the control samples) at each time point, indicating a strong influence of the WBE constituents on the microbial composition of the fecal sample. Notably, PMA treated samples followed the same trend as PMA-untreated samples.

Differences of the microbial communities (control vs. WBE incubations) were already visible after 30 min (t_0_), when the first sample was taken (Figure 1). This was in agreement with the observation, that many WBE compounds were already metabolized within the first minutes of incubation (see below). However, due to the long handling time under anoxic conditions (glove box), the first sampling could not be done earlier. The quick turnover and the rapid change in microbial composition indicated the immense activity of the microorganisms, and thus quality of the sample processing (no lag-phase) and experimental set-up.

By performing linear discriminant analysis coupled with effect size (LEfSe), we were able to identify 91 microbial taxa that were significantly increased or decreased under at least 4 conditions (concentration, time points) compared to the vehicle control. Table 1 shows all microbial taxa that either increased or decreased; Supplementary Table 4 includes also those taxa that showed a mixed behavior.

**Table 1:** Significantly increased and decreased microbial taxa.

**2**	**10**	**2**	**10**	**2**	**10**	**2**	**10**	**WBE conc., in mg/ml**						
**4 h**	**4 h**	**24 h**	**24 h**	**4 h**	**4 h**	**24 h**	**24 h**	**Time point**						
**PMA**				**DNA**				**Phylum**	**Class**	**Order**	**Family**	***Genus***	***Species***	**#OTU ID**
								Lentisphaerae	[Lentisphaeria]	Victivallales	Victivallaceae			577380
								Bacteroidetes	Bacteroidia	Bacteroidales	Bacteroidaceae	*Bacteroides*		583117
								**Bacteroidetes**	**Bacteroidia**	**Bacteroidales**	**Bacteroidaceae**	***Bacteroides***	***uniformis***	589071
								Bacteroidetes	Bacteroidia	Bacteroidales	S24-7			1108422
								Actinobacteria	Coriobacteriia	Coriobacteriales	Coriobacteriaceae			235127
								Bacteroidetes	Bacteroidia	Bacteroidales	Porphyromonadaceae	*Parabacteroides*		180082
								Bacteroidetes	Bacteroidia	Bacteroidales	[Barnesiellaceae]			315846
								Bacteroidetes	Bacteroidia	Bacteroidales	S24-7			321735
								Bacteroidetes	Bacteroidia	Bacteroidales	Porphyromonadaceae	*Parabacteroides*	*distasonis*	585914
								Bacteroidetes	Bacteroidia	Bacteroidales	S24-7			New_CleanUp_ ReferenceOTU2793
								Firmicutes	Bacilli	Turicibacterales	Turicibacteraceae	*Turicibacter*		New_ReferenceOTU583
								**Bacteroidetes**	**Bacteroidia**	**Bacteroidales**	**Bacteroidaceae**	***Bacteroides***		183480
								**Proteobacteria**	**Betaproteobacteria**	**Burkholderiales**	**Alcaligenaceae**	***Sutterella***		1974536
								Bacteroidetes	Bacteroidia	Bacteroidales	Bacteroidaceae	*Bacteroides*	*eggerthii*	349809
								Actinobacteria	Coriobacteriia	Coriobacteriales	Coriobacteriaceae	*Collinsella*	*aerofaciens*	363794
								Firmicutes	Clostridia	Clostridiales	Veillonellaceae	*Phascolarctobacterium*		916143
								Firmicutes	Clostridia	Clostridiales	Ruminococcaceae	*Ruminococcus*		195947
								Proteobacteria	Betaproteobacteria	Burkholderiales	Alcaligenaceae	*Sutterella*		2201995
								Proteobacteria	Deltaproteobacteria	Desulfovibrionales	Desulfovibrionaceae	*Bilophila*		359872
								Tenericutes	Mollicutes	RF39				569244
								Firmicutes	Erysipelotrichi	Erysipelotrichales	Erysipelotrichaceae			580008
								Firmicutes	Clostridia	Clostridiales	Veillonellaceae	*Veillonella*	*dispar*	585419
								Firmicutes	Clostridia	Clostridiales	Lachnospiraceae			791522
								Bacteroidetes	Bacteroidia	Bacteroidales	Bacteroidaceae	*Bacteroides*	*uniformis*	New_ReferenceOTU298
								Bacteroidetes	Bacteroidia	Bacteroidales	S24-7			New_ReferenceOTU416
								Firmicutes	Clostridia	Clostridiales	Ruminococcaceae	*Faecalibacterium*	*prausnitzii*	367433
								Firmicutes	Clostridia	Clostridiales	Ruminococcaceae			193778
								Firmicutes	Clostridia	Clostridiales	Ruminococcaceae			328544
								Firmicutes	Clostridia	Clostridiales	Ruminococcaceae			514523
								Firmicutes	Erysipelotrichi	Erysipelotrichales	Erysipelotrichaceae	*RFN20*		524633
								Firmicutes	Clostridia	Clostridiales	Ruminococcaceae			New_ReferenceOTU192
								Firmicutes	Clostridia	Clostridiales	Ruminococcaceae	*Faecalibacterium*	*prausnitzii*	New_ReferenceOTU431
								Firmicutes	Clostridia	Clostridiales	Ruminococcaceae			New_ReferenceOTU75
								Firmicutes	Clostridia	Clostridiales	Ruminococcaceae			176507
								Firmicutes	Clostridia	Clostridiales	Ruminococcaceae			361722
								Firmicutes	Clostridia	Clostridiales	Ruminococcaceae	*Faecalibacterium*	*prausnitzii*	370287
								**Firmicutes**	**Clostridia**	**Clostridiales**	**Ruminococcaceae**	***Faecalibacterium***	***prausnitzii***	514940
								Firmicutes	Clostridia	Clostridiales	Ruminococcaceae	*Faecalibacterium*	*prausnitzii*	851865
								Firmicutes	Clostridia	Clostridiales	Ruminococcaceae			New_ReferenceOTU140
								Firmicutes	Clostridia	Clostridiales	Ruminococcaceae			New_ReferenceOTU486
								Firmicutes	Clostridia	Clostridiales	Ruminococcaceae			182036
								Firmicutes	Clostridia	Clostridiales	Ruminococcaceae			187504
								Firmicutes	Clostridia	Clostridiales	Ruminococcaceae			291445
								Firmicutes	Clostridia	Clostridiales	Ruminococcaceae	*Ruminococcus*		362342
								Firmicutes	Clostridia	Clostridiales	Ruminococcaceae			367213

*Significantly increased (green) and decreased (red) microbial taxa (p < 0.05) under different conditions: WBE concentrations (2 and 10 mg WBE/ml HFS), timepoints (4 h, 24 h), compared to the vehicle control. Four left columns show results from PMA treated samples; the next four columns give the results from un-treated (DNA) samples. The full list of all 91 microbial taxa is given in Supplementary Table 4 Microbial taxa with abundance above 1% are printed bold and highlighted*.

**Figure 2:**
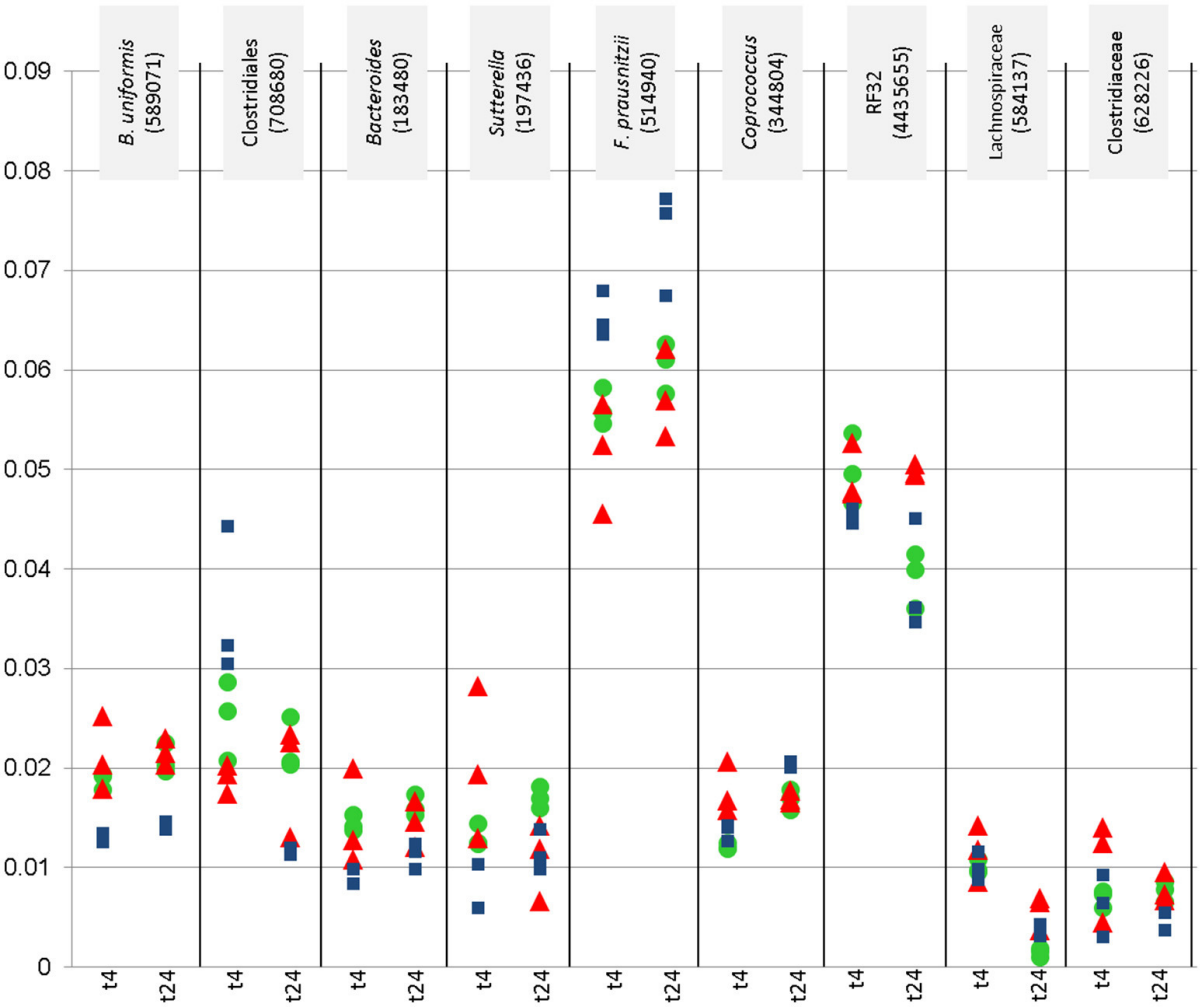
Relative abundance (y-axis) of most abundant microbial signatures (>1%), that showed significantly higher or lower counts under certain conditions (concentration, time point). This diagraph only shows PMA treated samples. Blue squares refer to vehicle samples (no added WBE), red triangles refer to 10 mg/ ml added WBE, green dots to 2 mg/ml.

Nine of these taxa appeared with a relative abundance of >1% in the datasets. Their abundance, and the effect of the WBE is displayed separately in Figure 2. One taxon of *Faecalibacterium prausnitzii* was found to be highly abundant in some samples (up to 9%).

Overall, the results from PMA-treated and untreated samples overlapped strongly, and DNA as well as PMA-treated samples gave the same trend of decrease and increase of certain taxa.

Signatures of Victivallaceae (Lentisphaerae), *Bacteroides* (in particular *Bacteroides uniformis* and *B. eggerthi*, Bacteroidetes*)*, S24-7 taxa (Bacteroidetes), Coriobacteriaceae (in particular *Collinsella aerofaciens*; Actinobacteria), Barnesiellaceae (Bacteroidetes), Parabacteroides (in particular *Parabacteroides distasonis*; Bacteroidetes*), Turicibacter* (Firmicutes; Bacilli), *Sutterella* (beta-proteobacteria, Alcaligenaceae) were found to be significantly more abundant in samples containing WBE than in samples without the extract, whereas taxa belonging to Ruminococcaceae (in particular *Faecalibacterium prausnitzii*) and Erysipelotrichaceae (RFN20) were obviously inhibited by WBE addition. Some Clostridiales/Lachnospiraceae taxa appeared to relatively decrease first and increase at the later time points of sampling, indicating a potential involvement in intermediate turnover.

### Addition of WBE has an effect on the function of fecal microbial communities *in vitro*

To obtain an overview on the functional capabilities of the microbial communities under different conditions, we performed PICRUSt analysis, estimating the functions based on 16S rRNA gene information (Langille et al., 2013, Table 2).

**Table 2:** Significantly increased and decreased microbial functions.

**Time point**	**4**	**4**	**24**	**24**	**4**	**4**	**24**	**24**	
**WBE concentration in mg/ml**	**2**	**10**	**2**	**10**	**2**	**10**	**2**	**10**	
**KEGG pathways**	**PMA**				**DNA**				
Function unknown									Stimualted by WBE
Carbonfixation pathways in prokaryotes									
Energy metabolism									
Lipopolysaccharide biosynthesis									
Lipopolysaccharide biosynthesis proteins									
Membrane and intracellular structural molecules									
Nucleotide excisionrepair									
Protein folding and associated processing									
Aminoacyl_tRNAbiosynthesis									
Chaperones and folding catalysts									
Citratecycle_TCAcycle_									
Folate biosynthesis									
General function prediction only									
Lipid biosynthesis proteins									
Lysosome									
Pyruvate metabolism									
Secretion system									
Transcription machinery									
Oxidative phosphorylation									partially stimulated/inhibited by WBE
Phenylalanine tyrosine and tryptophan biosynthesis									
Flagellar assembly									
DNA repair and recombination proteins									
Bacterial motility proteins									
Arginine and proline metabolism									
Purine metabolism									
RNA degradation									
Galactose metabolism									
Glycine_serine and threonine metabolism									
Nicotinate and nicotinamide metabolism									
Peptidases									
Starch and sucrose metabolism									
Thiamine metabolism									
Bacterial chemotaxis									
Two_component system									
Valine_leucine an disoleucine biosynthesis									
DNA replication proteins									
Glycero phospholipid metabolism									
Cysteine and methionine metabolism									
Aminosugar and nucleotide sugar metabolism									inhibited by WBE
Cytoskeleton proteins									
Pantothenate and CoA biosynthesis									
Ribosome Biogenesis									
ABC transporters									
Chromosome									
Sporulation									
Porphyrin and chlorophyll metabolism									
Transcriptionfactors									
Transporters									

*Significantly increased (green) and decreased (red) estimated microbial functions (p < 0.05) under different conditions (WBE concentration, time points), compared to the vehicle control. Four left columns show results from PMA treated samples, the next four columns give the results from untreated (DNA) samples*.

Fifty-one KEGG pathways were identified that showed a significantly (*p* < 0.05) different abundance compared to the vehicle control (Table 2). Pathways belonging to energy metabolism (carbon fixation pathways in prokaryotes, energy metabolism) appeared to be stimulated, as well as glycan biosynthesis and metabolism (lipopolysaccharide biosynthesis), carbohydrate metabolism (citrate_TCA cycle, pyruvate metabolism), metabolism of cofactors and vitamins (folate biosynthesis) and other general cellular processes. Other pathways appeared to be partially stimulated and inhibited, such as oxidative phosphorylation (energy metabolism) or metabolism of different amino acids. Notably, metabolic pathways, such as galactose metabolism, starch and sucrose metabolism were found to be stimulated only in non-PMA treated samples.

### Incubation with HFS leads to intensive metabolization of WBE constituents *in vitro*

All in all, from the processed LC-HRMS data (Supplementary Tables 1–3), 58 compounds were annotated, that significantly increased or decreased over time and that were not contained in the fecal matrix samples, indicating that they were either WBE constituents or metabolites thereof (Table 3). Many compounds were tentatively annotated by comparison of their monoisotopic mass, molecular formula and fragmentation data with literature data (details: Supplementary Data Sheet 1; MSI identification level 2–3^2^ Some compounds were unambiguously identified by comparison with authentic reference compounds (MSI identification level 1).

**Table 3:** Identified compounds significantly changing over incubation time.

**Peak #**	**Compound name**	**RT (min)**	**Monoisotopic mass**	**Molecular formula**	**Δ (ppm)**	**Human fecal suspension**		**PBS**	**Literature/database**
						**WBE (2 mg/ml)**	**WBE (10 mg/ml)**	**WBE (10 mg(/ml)**	
1	saccharose^b^	1.33	342.1166	C_12_H_22_O_11_	4.14				
2	malic acid^b^	1.43	134.0203	C_4_H_6_O_5_	−1.35				https://metlin.scripps.edu/^b^
3	citric acid^a^	1.66	192.0263	C_6_H_8_O_7_	1.94				
4	succinic acid^a^	1.96	118.0252	C_4_H_6_O_4_	−2.42				
5	isopropyl maleate^b^	2.65	158.0565	C_7_H_10_O_4_	−2.00				http://www.hmdb.ca/^b^
6	protocatechuic acid^a^^+^	4.93	154.0252	C_7_H_6_O_4_	−2.32				Goodrich and Neilson, 2014^+^
7	(epi)gallocatechin^b^^#^	5.17	306.0742	C_15_H_14_O_7_	5.12				https://metlin.scripps.edu/^b^; Jürgenliemk et al., 2007^#^
8	catechol^b^	5.62	110.0352	C_6_H_6_O_2_	−2.99				https://metlin.scripps.edu/^b^
9	vanillic acid hexoside^b^^#^	6.08	330.0957	C_14_H_18_O_9_	5.26				Liao et al., 2014; Ammar et al., 2015^b^
10	salicin^a^^#^	6.72	286.1055	C_13_H_18_O_7_	3.78				Agnolet et al., 2012^#^; Kammerer et al., 2005^#^
11	cysteine-saligenin-adduct^b^	6.98	227.0611	C_10_H_13_O_3_NS	1.94				
12	saligenin^a^^#^	7.06	124.0512	C_7_H_8_O_2_	−1.51				Kammerer et al., 2005^#^
13	5-(3',4',5'-trihydroxyphenyl)-γ-valerolactone^b^^+^	7.68	224.0677	C_11_H_12_O_5_	1.26				Takagaki and Nanjo, 2010^+^
14	gentisic acid^a^	7.70	154.0252	C_7_H_6_O_4_	−2.25				
15	4-hydroxybenzoic acid^a^	7.72	138.0301	C_7_H_6_O_3_	−3.51				
16	4-hydroxy-5-(dihydroxyphenyl)valeric acid^b^^+^	8.88	226.0834	C_11_H_14_O_5_	1.60				Takagaki and Nanjo, 2013^b^^+^
17	dihydrocaffeic acid^a^^+^	9.13	182.0570	C_9_H_10_O_4_	2.01				Monagas et al., 2010^+^; Takagaki and Nanjo, 2013^+^
18	(epi)catechin-(epi)catechin^b^^#^	9.23	578.1430	C_30_H_26_O_12_	2.82				https://metlin.scripps.edu/^b^, Jürgenliemk et al., 2007^#^
19	catechin^a^^#^	9.62	290.0793	C_15_H_14_O_6_	4.59				Jürgenliemk et al., 2007^#^
20	4-oxo-5-(dihydroxyphenylvaleric)acid^b^^+^	9.79	224.0677	C_11_H_12_O_5_	1.26				Takagaki and Nanjo, 2013^b^^+^
21	dihydroxybenzoic acid^b^	9.87	154.0251	C_7_H_6_O_4_	−2.37				https://metlin.scripps.edu/^b^
22	hydroxy(iso)caproic acid^b^^+^	10.52	132.0771	C_6_H_12_O_3_	−3.74				http://www.hmdb.ca^b^; Zheng et al., 2011^+^
23	acetylsalicin^b^^#^	10.54	328.1161	C_15_H_20_O_8_	3.82				Yang et al., 2013^#^; Kammerer et al., 2005^b^^#^
24	hydroxy(iso)caproic acid^+^	10.88	132.0771	C_6_H_12_O_3_	−3.68				http://www.hmdb.ca^b^; Zheng et al., 2011^+^
25	chlorogenic acid^a^^#^	10.92	354.0955	C_16_H_18_O_9_	4.48				Zaiter et al., 2016^#^
26	(epi)catechin-(epi)catechin-(epi)catechin^b^^#^	10.95	866.2067	C_45_H_38_O_18_	2.47				Jürgenliemk et al., 2007; Bijttebier et al., 2016^b^; Piccinelli et al., 2016; Zaiter et al., 2016^#^
27	syringin^b^^#^	11.38	372.1421	C_17_H_24_O_9_	4.44				Agnolet et al., 2012^#^; Tótha et al., 2016^b^
28	5-(dihydroxyphenyl)-y-valerolactone^b^^+^	12.34	208.0726	C_11_H_12_O_4_	0.07				Goodrich and Neilson, 2014^b^^+^; Takagaki and Nanjo, 2013
29	benzyl-hexoside-pentoside^b^	12.40	402.1539	C_18_H_26_O_10_	4.50				Delgado De La Torre et al., 2015^b^
30	ampelopsin^b^^#^	12.42	320.0533	C_15_H_12_O_8_	4.16				Agnolet et al., 2012^#^; Yang et al., 2012^b^
31	diyhdroquercetin sulfate^b^	12.83	314.0156	C_15_H_12_O_10_S	1.56				Vacek et al., 2013^b^
32	benzyl-hexoside-pentoside^b^	12.89	402.1541	C_18_H_26_O_10_	4.95				Delgado De La Torre et al., 2015^b^
33	acetylsalicin^b^^#^	14.39	328.1159	C_15_H_20_O_8_	3.42				Kammerer et al., 2005^b^^#^
34	3-(3-hydroxyphenyl)propionic acid^a^^+^	14.65	166.0616	C_9_H_10_O_3_	−1.88				Takagaki and Nanjo, 2013^+^
35	4-hydroxy-5-(hydroxyphenyl))valeric acid^b^^+^	16.61	210.0882	C_11_H_14_O_4_	0.55				Takagaki and Nanjo, 2013^b^^+^
36	dihydroquercetin^a^^#^	17.78	304.0585	C_15_H_12_O_4_	4.62				Agnolet et al., 2012^#^
37	(+)-naringenin-5-glucoside^b^^#^	17.89	434.1215	C_21_H_22_O_10_	3.64				Kammerer et al., 2005^#^
38	salicylic acid^a^^#^	17.99	138.0302	C_7_H_6_O_3_	−3.29				Kammerer et al., 2005^#^
39	rosarin^b^	18.34	428.1683	C_20_H_28_O_10_	3.08				Tolonen et al., 2003^b^
40	salicortin^b^^#^	18.49	424.1371	C_20_H_24_O_10_	1.79				Kammerer et al., 2005^b^^#^
41	(-)-naringenin-5-glucoside^b^^#^	18.84	434.1215	C_21_H_22_O_10_	3.09				Kammerer et al., 2005^b^^#^
42	5-(dihydroxyphenyl)valeric acid^b^^+^	19.65	210.0882	C_11_H_14_O_4_	0.31				Takagaki and Nanjo, 2013^b^^+^
43	cinnamyl-(6‘-O- xylopyranosyl)-O-glucopyranoside^b^	19.66	428.1690	C_20_H_28_O_10_	2.94				Tolonen et al., 2003^b^
44	hyperoside^a^^#^	20.70	464.0957	C_21_H_20_O_12_	4.27				Nybakken and Julkunen-Tiitto, 2013^#^
45	cinnamyl-hexoside-deoxyhexoside^b^	20.95	442.1840	C_21_H_30_O_10_	2.80				https://metlin.scripps.edu/^b^
46	dihydrocinnamylhexoside-deoxyhexoside^b^	21.52	444.2001	C_21_H_32_O_10_	3.42				Tolonen et al., 2004^b^
47	naringenin-7-glucoside^a^^#^	21.90	434.1215	C_21_H_22_O_10_	1.58				Kammerer et al., 2005^#^
48	salireposide^b^^#^	22.34	406.1268	C_20_H_22_O_9_	3.28				Kammerer et al., 2005^b^^#^
49	phenylpropionic acid^b^^+^	24.15	150.067	C_9_H_10_O_2_	−0.35				http://www.hmdb.ca^b^; Rechner et al., 2004; Goodrich and Neilson, 2014; Orrego-Lagarón et al., 2016^+^
50	acetylsalicortin^b^^#^	24.79	466.1475	C_22_H_26_O_11_	1.86				Kammerer et al., 2005^b^^#^
51	grandidentatin/isograndidentatin^b^^#^	24.97	424.173	C_21_H_28_O_9_	4.23				Yang et al., 2013^#^; Jervis et al., 2015^b^
52	isosalipurposide^b^^#^	25.40	434.1211	C_21_H_22_O_10_	3.99				Kammerer et al., 2005^b^^#^
53	grandidentatin/isograndidentatin^b^^#^	25.46	424.174	C_21_H_28_O_9_	4.02				Yang et al., 2013^#^; Jervis et al., 2015^b^
54	hydroxyphenylvaleric acid^b^^+^	25.87	194.093	C_11_H_14_O_3_	0.00				Takagaki and Nanjo, 2013^b^^+^
55	benzoylsalicin^b^^#^	27.51	390.132	C_20_H_24_O_9_	3.07				Kammerer et al., 2005^b^^#^
56	naringenin^a^^#^	30.77	272.0683	C_15_H_12_O_5_	4.58				Freischmidt et al., 2012^#^
57	HCH-acetylsalicortin^b^^#^	32.46	604.1893	C_29_H_32_O_14_	2.45				Merken and Clausen, 1992^#^; Keefover-Ring et al., 2014^b^
58	tremulacin^b^^#^	35.54	528.163	C_27_H_28_O_11_	1.73				Kammerer et al., 2005^b^^#^

Compounds **1**–**58** were undetectable in the vehicle control samples. Therefore, they can be assumed to be derived from willow bark extract or its metabolites.

a Identified by comparison with authentic reference compound.

b Tentative identification based on monoisotopic mass and comparison with fragmentation patterns in literature or databases (HMDB, Metlin, Massbank, mzCloud; for details, see Supplementary Data Sheet 1); structural isomers cannot be ruled out;

# Described in the literature in Salix sp;

+ Described in the literature as intestinal/fecal metabolite.

Color code:

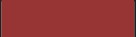
 Decreasing over 24 h (ratio t0/t24 < 0.5, p < 0.05).

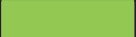
 Newly formed or increasing over 24 h (ratio t0/t24 >2, p < 0.05).

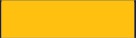
 Intermediate (ratio t0/t4 > 2, p < 0.05; ratio t4/t24 > 1.25).

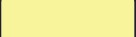
 No significant change over 24 h.

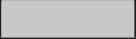
 Not detectable.

**Figure 3:**
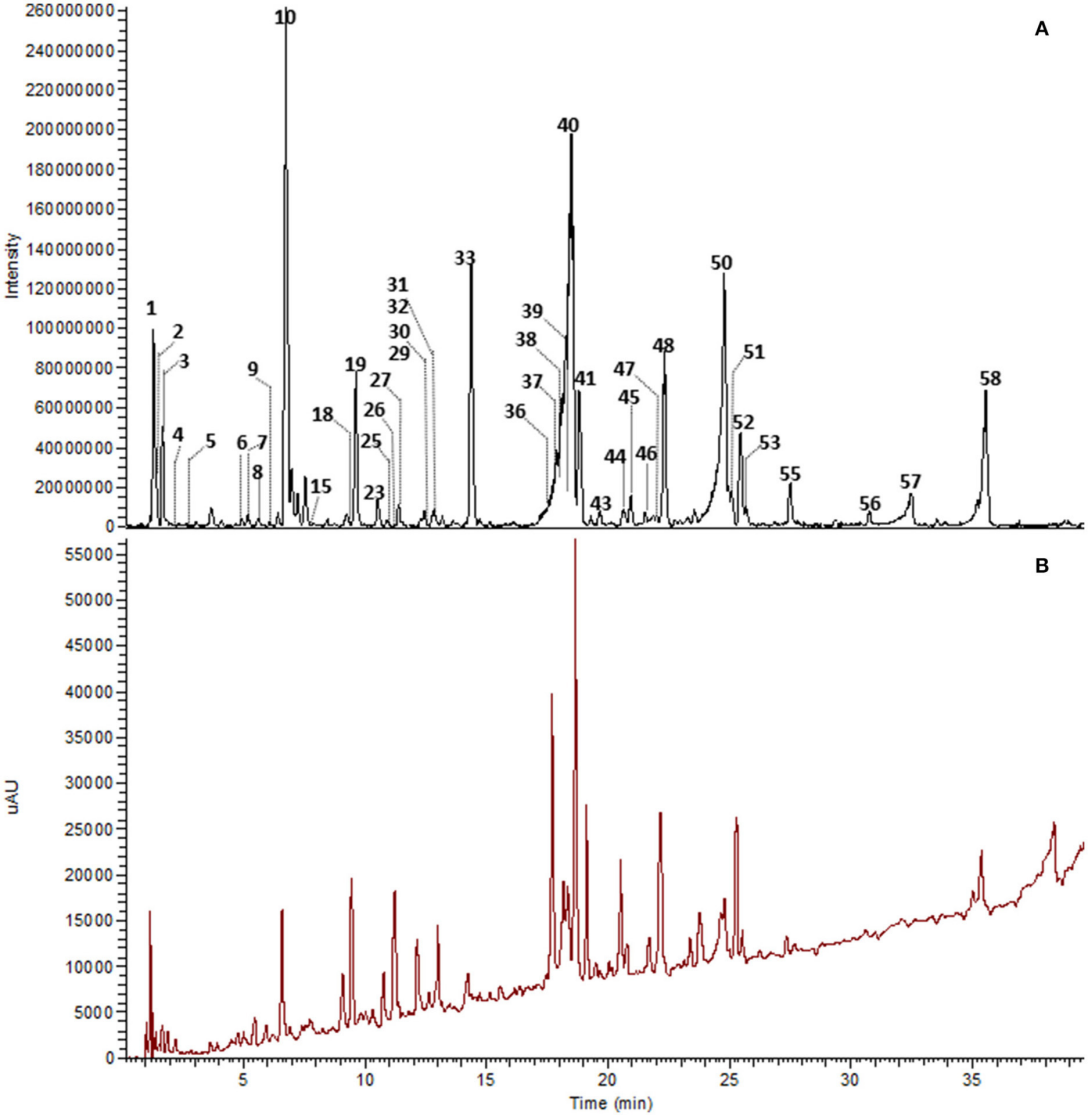
UHPLC-DAD-HRMS chromatogram of WBE (10 mg/ml) in PBS buffer at t0. Peak numbers as indicated in Table 3 and Supplementary Data Sheet 1. **(A)** Background subtracted base peak chromatogram, ESI negative mode, m/z 100–1500. **(B)** DAD total scan, 220–400 nm.

**Figure 4:**
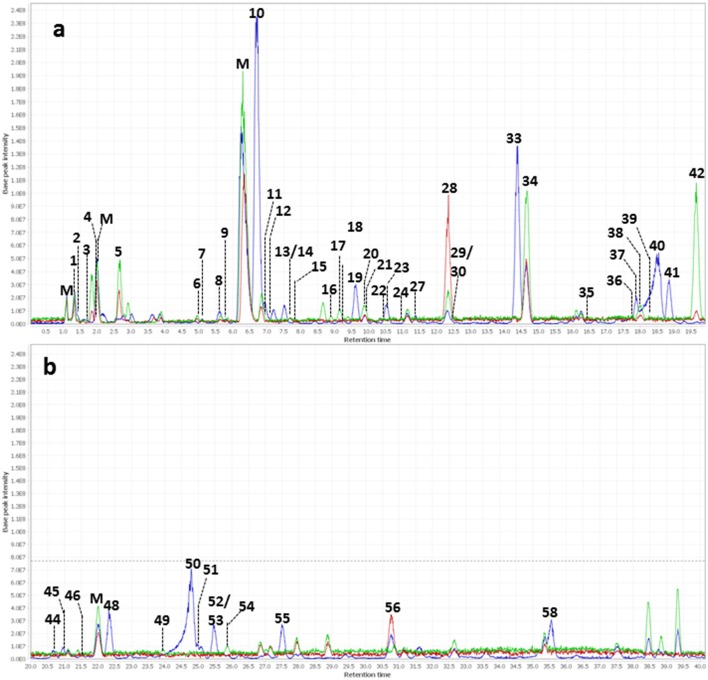
Overlay of background subtracted base peak chromatograms (m/z 100–1500) of WBE (2 mg/ml) in HFS (0.1 g/ml in PBS buffer) at t_0_ (blue) t_4_ (red) and t_24_ (green). **(a)** Chromatogram at retention time 0–20 min; **(b)** chromatogram at retention time 20–40 min. Peak numbers are as indicated in Table 3. M, matrix peaks (peaks that are also contained in HFS samples without WBE addition).

The major constituents detected in unmetabolized WBE are salicylic alcohol derivatives, flavonoids and catechin, together with minor amounts of aliphatic and aromatic acids, aromatic alcohols and procyanidins (Figure 3, Table 3, Supplementary Data Sheet 1). Most of these compounds were found to readily degrade upon incubation with HFS (Figure 4), while almost all of them were found to be stable over 24 h when incubated in PBS buffer under the same conditions (Supplementary Table 3), indicating that the observed reactions are due to metabolization by the microbes present in HFS.

Regarding salicyl alcohol derivatives, we were able to detect salicin (**10**), acetylsalicin (**23**), acetylsalicin isomer (**33**), salicortin (**40**), salireposide (**48**), acetylsalicortin (**50**), benzoylsalicin (**55**), 1-hydroxy-6-oxo-2-cyclohexenecarboxylate (HCH)- acetylsalicortin (**57**), and tremulacin (**58**). Interestingly, the levels of compound **23** (acetylsalicin) significantly increased upon incubation in PBS buffer alone over 24 h, while its isomer (**33**) slightly decreased (Supplementary Table 3). Also other related salicylic alcohol derivatives, acetylsalicortin (**50**) and HCH-acetylsalcortin (**57**) were found to slightly decrease over time when incubated with PBS buffer alone. Therefore, we assume that the increase of compound **23** is on one hand due to the cleavage of HCH moieties in **50** and **57**, and on the other hand due to the rearrangement of the acetyl group in **33**. The other salicyl alcohol derivatives were stable during the whole incubation period in PBS buffer. Upon incubation with HFS, however, they were readily metabolized within 24 h (Table 3). Interestingly, those salicylic alcohol derivatives that contained a HCH moiety, i.e., compounds **40**, **50**, **57** and **58**, were metabolized particularly fast upon HFS-treatment. The levels of compounds **40**, **50** and **58** were already reduced at t0, and **57** was not even detectable in the t0 sample of WBE 2 mg/ml incubated with HFS due to its obviously fast metabolization. This type of compounds has been described in the literature to possess low stability and to be more easily degraded in the presence of enzymes than salicylic alcohol derivatives possessing no HCH moiety (Julkunen-Tiitto, 1992), (Ruuhola et al., 2003). This might explain the reduced stability of **50** and **57** under the applied experimental conditions, but also the extremely fast metabolization observed for this compound type upon HFS addition.

Also the flavonoids detected in WBE were readily metabolized upon incubation with HFS. The minor flavonoids ampelopsin (**30**), dihydroquercetin (**36**), and hyperoside (**44**) significantly decreased over incubation time in HFS. Compound **31** was tentatively annotated to dihydroquercetin sulfate, a compound so far undescribed in herbal sources. **31** was obviously even metabolized that fast that it was undetectable in both WBE concentrations incubated with HFS already at t0. Naringenin glycosides (**37, 41, 47**) that constitute the major flavonoids in WBE were also readily metabolized at both WBE concentrations. Their aglycone naringenin (**56**) was present in unmetabolized WBE only at low levels. Upon incubation of both WBE concentrations with HFS, naringenin levels transiently increased, obviously due to microbial hydrolysis of the respective glycosides. Formed naringenin was obviously further metabolized since its concentration decreased again between 4 and 24 h of incubation.

From the group of flavan-3-ols, we could detect catechin (**19**) as a major constituent of the studied WBE, together with low levels of dimeric and trimeric procyanidins (**18, 26**) and (epi)gallocatechin (**7**). The dimeric procyanidin (**18**) significantly decreased upon incubation of both WBE concentrations with HFS, the trimeric procyanidin (**26**) obviously degraded that fast that it was already undetectable at t0 in HFS-incubated samples. Catechin (**19**) and (epi)gallocatechin (**7)** were significantly reduced over time in WBE 2 mg/ml in HFS, and slightly but insignificantly reduced in WBE 10 mg/ml in HFS.

Further constituents detected in WBE belonged to the classes of aliphatic acids (**2, 3, 4**), aromatic acid derivatives (**9, 25, 51, 53**) and aromatic alcohol derivatives (**27, 29, 32, 39, 43, 45, 46**). Some of these compounds were detected in WBE for the first time.

Concerning aromatic acids, compound **9** was tentatively identified as vanillic acid hexoside. Vanillic acid 4-β-D-glucoside has already been isolated from the leaves of *S. matsudana* (Liao et al., 2014), but has not yet been described in commercial willow bark extracts. Compound **25** was identified as chlorogenic acid. Compounds **51** and **53** were tentatively identified as the coumaroyl-dihydroxycyclohexyl-glycosides grandidentatin and isograndidentatin, that have been isolated from *Salix pseudolasiogyne* twigs (Yang et al., 2013). For compounds **51** and **53**, a slight decrease could also be observed in the PBS control. Therefore, it seems that these compounds are somewhat unstable at the applied experimental conditions.

Concerning aromatic alcohols, compound **27** was tentatively assigned to the sinapylalcohol glycoside syringin that has been detected in different commercial willow bark preparations (Agnolet et al., 2012). Compounds **29** and **32** were tentatively assigned to benzyl alcohol glycosides bearing a hexose-pentose moiety that have not yet been found in *Salix* sp. to the best of our knowledge. Furthermore, we detected a series of cinnamylalcohol derivatives so far undescribed in *Salix* sp.: Compounds **39** and **43** were tentatively identified as cinnamyl-hexoside-pentosides, compound **45** as cinnamyl-hexoside-deoxyhexoside, and compound **46** as dihydrocinnamyl-hexoside-deoxyhexoside. Since cinnamylalcohol derivatives are major constituents in roseroot (*Rhodiola rosea* L.), we used an ethanolic roseroot extract as reference, and were able to assign compound **39** to rosarin, and compound **43** to cinnamoyl-(6′-O-xylopyranosyl)-O-glucopyranoside (Tolonen et al., 2003).

Also these minor WBE constituents degraded over time upon incubation in HFS. Compounds **25**, **32** and **43** degraded that fast that they were already undetectable in the t0 samples.

On the other hand, numerous compounds were found to be increased in their levels or newly formed upon incubation of WBE with HFS. Many putative microbial metabolites like saligenin (**12**) were undetectable in unmetabolized WBE, but already detectable at t0 in HFS-incubated WBE samples. Obviously, 30 min of incubation are time enough for the first metabolic reactions to occur.

Compounds **5, 6** (protocatechuic acid)**, 11** (saligenin-cysteine adduct)**, 12** (saligenin)**, 14** (gentisic acid)**, 15** (4-hydroxybenzoic acid)**, 21** (dihydroxybenzoic acid) and **38** (salicylic acid) significantly increased at both WBE concentrations, whereas for compounds **16** [4-hydroxy-5-(dihydroxyphenyl)valeric acid], **22, 24, 34** [3-(3′-hydroxyphenyl)propionic acid], **42** [5-(dihydroxyphenyl)valeric acid] and **54 (**hydroxyphenylvaleric acid), the increase was only significant at WBE 2 mg/ml, and for compound **17** (dihydroxyphenylpropionic acid) at WBE 10 mg/ml. Compounds **20** [4-oxo-5-(dihydroxyphenyl)valeric acid], **35** [4-hydroxy-5(-hydroxyphenyl)valeric acid] and **49** (phenylpropionic acid) were only formed at incubation with WBE 2 mg/ml and undetectable in HFS samples incubated with WBE 10 mg/ml. The levels of **13** (5-trihydroxyphenyl- γ-valerolactone)**, 28** (dihydroxyphenyl-γ-valerolactone) and **56** (naringenin) were found to increase between t0 and t4 and to subsequently decrease again. Therefore, they were regarded as intermediates that are further decomposed upon formation.

## Discussion

### Addition of WBE supports beneficial microorganisms specialized in complex carbohydrate degradation *in vitro*

Amongst others, in particular Bacteroidales signatures, including *Bacteroides* (*B. uniformis, B. eggerthi*), S24-7 taxa, Barnesiellaceae, *Parabacteroides* (*P. distasonis*) increased under WBE addition to the fecal sample used for the study. *Bacteroides* species have a fundamental role in the gut ecosystems, fermenting simple and complex carbohydrates, based on a sophisticated polysaccharide degradation machinery and glucosidase activity (Xu et al., 2003). Overall, *Bacteroides* are considered beneficial microorganisms, stimulating e.g., the gut lining to produce fucosylated glycans, ameliorating metabolic and immunological dysfunction in mice with induced obesity and supporting angiogenesis (formation of blood vessels) in newborns (Coyne et al., 2005; Cano et al., 2012). *B. uniformis* and *P. distasonis* were found to be able to cleave the O-glycosidic linkages of different flavonoids (Braune and Blaut, 2016), like flavonol glycosides (Bokkenheuser et al., 1987) and flavanone glycosides (Miyake et al., 1997). Therefore, these taxa might be involved in the observed metabolization of flavonoid glycosides present in WBE. *Bacteroides uniformis* was recently discussed to be involved in isoflavone degradation, whereas *B. eggerthi* was proposed to be involved in glycitein/genistein metabolism, although, functional details remained largely hidden (Renouf and Hendrich, 2011).

Bacteroidales lineage S24-7 (“*Candidatus* Homeothermaceae”) remains currently without cultivated representatives. However, genome-based analysis revealed a complex genetic capability to ferment a variety of carbohydrates, such as alpha-glucans, complex plant cell wall glycans, or even host-derived glycans (Ormerod et al., 2016).

Saccharolytic activity toward mono- and disaccharides, as well as metabolisation of sugar alcohols and sugar acids was reported for Victivallaceae as well (Hedlund et al., 2004), which were also found to increase upon WBE addition to the fecal sample used in our study. The same behavior was found for Coriobacteriaceae that are obviously involved in the activation of dietary polyphenols (Clavel et al., 2014), as their abundance increased in a study with rats under wild blueberry diet (Lacombe et al., 2013). Members of Coriobacteriaceae have been identified to be involved in the reduction, dehydroxylation and C-ring cleavage of flavone compounds, and thus might have been involved in key metabolic processes in our study (Braune and Blaut, 2016).

Although much other information is currently missing, and the detailed functions of each involved microorganism need to be enlightened by other means such as transcriptomics or stable isotope probing, we can argue, that the identified microbial taxa that increased under WBE influence are excellent candidates for the degradation of the substrate provided. Notably, Ruminococcaceae, and in particular *Faecalibacterium prausnitzii*, was negatively affected by WBE. *Faecalibacterium prausnitzii* is one of the most abundant bacteria in the gut of healthy adults (more than 5% of the total bacterial population; Tap et al., 2009; Walker et al., 2011). *Faecalibacterium prausnitzii* is considered to be a beneficial microorganism, as it synthesizes butyrate and short-chain fatty acids through acetate metabolism and the fermentation of dietary and host carbohydrates, such as fructose, pectin, starch, N-acetylglucosamine etc. (Miquel et al., 2014). In the specific case of our WBE experiments, *F. prausnitzii* signatures were found to be decreased compared to control. We can conclude that *F. prausnitzii* might either be out-competed by Bacteroides, is incapable of metabolizing WBE compounds, or is even inhibited by certain constituents of the extract. Specific experiments with cultures of *F. prausnitzii* could be performed, in order to elucidate a possible, potentially concentration-dependent inhibition. However, it shall be mentioned, that *F. prausnitzii* signatures were found also in PMA-treated samples, indicating an intact cell wall, and thus, activity of *F. prausnitzii* cannot be excluded, considering also the high abundance of its signatures found in our samples (up to 9%).

### Proposed *in vitro* metabolization pathways of main WBE compound classes

#### Salicylic alcohol derivatives

Salicylic alcohol derivatives are major WBE constituents that are known to be converted to salicylic acid after oral administration and are thought to play an important role in the extract's clinically proven efficacy against low back pain. However, plasma salicylic acid levels reached after WBE ingestion are comparably low. Therefore, salicylic alcohol derivatives alone cannot explain the overall efficacy of willow bark preparations, and other WBE constituents are supposed to be involved in the pharmacological effects of WBE (Schmid et al., 2001; Committee on Herbal Medicinal Products, 2009).

In our *in vitro* experiment, saligenin (**12**), the common aromatic alcohol of all salicylic alcohol derivatives except salireposide, was not detectable in unmetabolized WBE, but its levels significantly increased over incubation time with HFS. Next to saligenin, we could tentatively annotate another related metabolite, namely a cysteine-saligenin conjugate (**11**) that almost co-eluted with saligenin. The MS signal of both compounds **11** and **12** was rather weak but their DAD signal was very pronounced, indicating that they are major metabolites of salicylic alcohol derivatives. The formation of cysteine adducts by intestinal bacteria is not commonly described. Quite the contrary, many enteric bacteria are known to possess cysteine S-conjugate β-lyase activity that allows them to cleave cysteine S-conjugates of xenobiotics secreted into the bile (Cooper et al., 2011). Taking into consideration that cysteine is a constituent of the anaerobic PBS buffer used for our experiments, we performed a control experiment, the results of which indicated that the observed saligenin-cysteine adduct is formed directly and without the involvement of fecal bacteria. Therefore, **11** has to be regarded as an artifact (further details are given in Identification details for compound 11 and Figure S1).

Apart from saligenin and its cysteine conjugate, salicylic acid (**38**) and gentisic acid (**14**) are further putative salicylic alcohol metabolites, which we could detect at low levels.

Taking these data together, we propose the following degradation for salicylic alcohol derivatives in our *in vitro* experimental setting: in a first step, the sugar, benzoyl and HCH moieties are cleaved by hydrolytic reactions. The major part of the resulting saligenin reacts to a cysteine-saligenin adduct that has to be regarded as an experimental artifact. The minor part is further metabolized to salicylic acid and gentisic acid, respectively We could not observe any metabolites potentially resulting from the HCH moiety present in some salicylic alcohol derivatives, like hydroxyl-cyclohexenone or catechol (Ruuhola et al., 2003). In fact, we tentatively annotated catechol (**8**) already in unmetabolized WBE. During incubation of WBE with HFS, the levels of the compound did not significantly change.

In early *in vitro* studies using intestinal sections of normal and antibiotic-treated rats, Fötsch and Pfeifer found that intestinal bacteria are able to degrade salicin to saligenin (Fötsch and Pfeifer, 1989). However, in *in vivo* pharmacokinetics studies in humans, the main salicin metabolite salicylic acid was already detectable in serum 1 h after oral administration of salicin or WBE, suggesting that salicin is obviously already absorbed in the stomach or upper intestinal tract and hydrolyzed before or during absorption (Schmid et al., 2001; Knuth et al., 2013). Similar results were obtained when the salicin derivative salicortin was orally administered to rats (Knuth et al., 2013).

This would mean that *in vivo*, salicyl alcohol derivatives could be cleaved and absorbed before reaching the colon. Therefore, the hydrolysis by intestinal bacteria observed in our study may not be relevant under physiological conditions. In plasma, the main metabolite of salicyl alcohol derivatives is salicylic acid, together with minor amounts of salicyluric acid and gentisic acid (Schmid et al., 2001). In addition, catechol sulfate was observed as a salicortin metabolite in serum (Knuth et al., 2013).

In our study, we could observe the formation of saligenin (**12**) and of the oxidative saligenin metabolites gentisic acid (**14**) and salicylic acid (**38**) when WBE was incubated with the fecal sample used in the experiments. This indicates that intestinal bacteria are capable to transform salicylic alcohol derivatives to these metabolites, provided that they reach the lower intestinal tract which might not be the case *in vivo*.

#### Flavonoids

**Figure 5:**
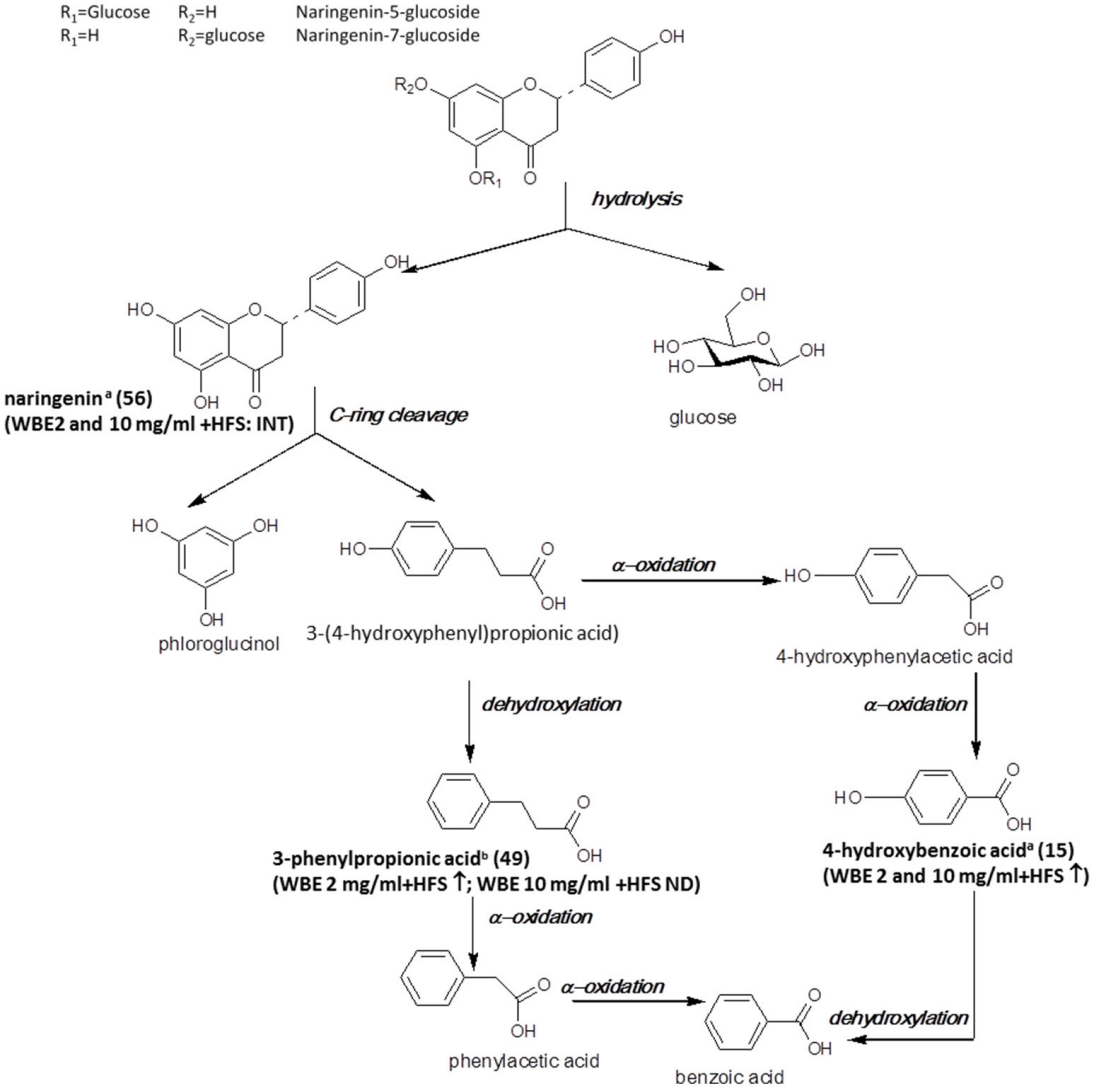
Microbial metabolism of naringenin glycosides as described in the literature (Rechner et al., 2004; Mosele et al., 2015; Orrego-Lagarón et al., 2016). Metabolites detected in the present study are printed in bold (a: identified by comparison with authentic reference compound; b: tentatively identified on basis of HRMS, molecular formula and fragmentation pattern; INT: intermediate; ↑: significantly increasing over time (ratio t24/t0 > 2, *p* < 0.05); ↓: significantly decreasing over time (ratio t24/t0 < 0.5; *p* < 0.05); ↔ no significant change over time).

The major flavonoids detected in the studied WBE are naringenin derivatives. Naringenin and its glycosides have been found to be readily metabolized by intestinal bacteria *in vitro* (Rechner et al., 2004; Pereira-Caro et al., 2015), in mice (Orrego-Lagarón et al., 2016) and in humans (Pereira-Caro et al., 2014). In Figure 5, the microbial metabolism of naringenin glycosides hitherto known is summarized, and metabolites detected in the present study are indicated.

In our study, naringenin (**56**) was formed upon incubation with HFS and subsequently further decomposed. The observed decrease of naringenin levels between t_4_ and t_24_ was much more pronounced for WBE 2 mg/ml (708-fold) than for WBE 10 mg/ml (1.56-fold) (Supplementary Tables 1, 2). A possible explanation for this phenomenon is that too high concentrations of certain WBE constituents or metabolites may inhibit bacterial species responsible for certain metabolization reactions. For example, Duda-Chodak investigated the impact of different flavonoid glycosides and aglycones on selected gut microbial species. They found that some flavonoid aglycones, among them naringenin, significantly inhibited bacterial growth, while, interestingly, the flavonoid glycosides had no negative impact. This might explain the retarded further metabolism of naringenin in WBE 10 mg/ml in contrast to WBE 2 mg/ml incubated with HFS, indicating that too high concentrations of formed naringenin might inhibit certain bacterial taxa involved in its further metabolism.

According to the literature, the further metabolism of naringenin involves cleavage of ring C, thereby leading to formation of 3-(4-hydroxyphenyl)propionic acid and phloroglucinol (Figure 5). However, in our study, we could detect neither of these two metabolites. 3-(4-Hydroxy-phenyl)propionic acid has been detected as intestinal microbial naringenin metabolite *in vitro* (Rechner et al., 2004; Pereira-Caro et al., 2015) and in mice (Orrego-Lagarón et al., 2016), while phloroglucinol is hardly ever recovered as final metabolite, since it obviously can be degraded into acetate, butyrate and CO_2_ (Brune and Schink, 1992; Possemiers et al., 2011). The reason why 3-(4-hydroxyphenyl)propionic acid was not detectable in our study is most likely due to the weak ionization of the compound under the applied ESI-MS conditions. Therefore, we might have missed low amounts of the compound due to insufficient sensitivity.

**Figure 6:**
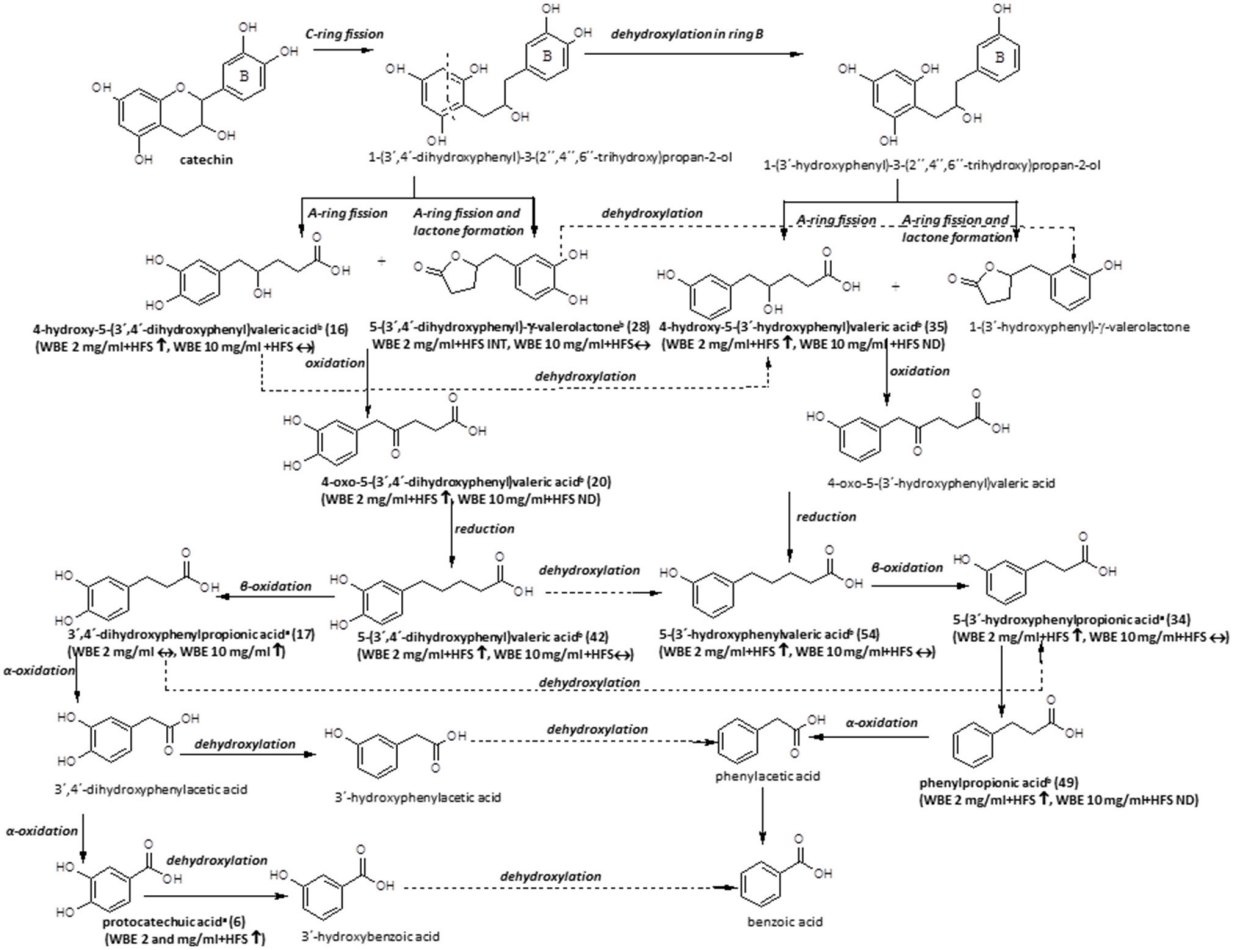
Microbial metabolism of catechin as described in the literature (Appeldoorn et al., 2009; Monagas et al., 2010; Takagaki and Nanjo, 2013). Metabolites detected in the present study are printed in bold (a: identified by comparison with authentic reference compound; b: tentatively identified on basis of HRMS, molecular formula and fragmentation pattern; WBE, willow bark extract; HFS, human fecal suspension; INT, intermediate; ↑, significantly increasing over time (ratio t24/t0 > 2, *p* < 0.05); ↓: significantly decreasing over time (ratio t24/t0 < 0.5; *p* < 0.05); ↔: no significant change over time).

According to the literature, 3-(4-hydroxyphenyl)propionic acid is further degraded to 3-phenylpropionic acid by dehydroxylation (Rechner et al., 2004; Pereira-Caro et al., 2015; Orrego-Lagarón et al., 2016). Indeed, this metabolite (**49**) was formed over time in WBE 2 mg/ml, but was not detectable in WBE 10 mg/ml incubated with HFS. However, it has to be considered that **49** might also arise from metabolism of flavan-3-ols present in WBE (Figure 6).

4-Hydroxybenzoic acid (**15**) was found to significantly increase in WBE 2 and 10 mg/ml incubated with HFS and might be formed by subsequent α-oxidations of 3-(4-hydroxyphenyl)propionic acid (Mosele et al., 2015). The compound was only detectable at trace levels.

#### Catechin derivatives

The microbial metabolism of catechins and procyanidins is quite well- described in the literature. A summary of known microbial degradation pathways and a comparison with data obtained in this study is provided in Figure 6.

One main compound that could be putatively attributed to the metabolism of catechin derivatives known so far was dihydroxyphenyl-γ-valerolactone (**28**). In WBE 2 mg/ml incubated with HFS, its levels initially increased but subsequently decreased again, indicating its further microbial conversion. 5-(3′,4′-Dihydroxyphenyl)-γ-valerolactone has been described to be formed during microbial degradation of (+)-catechin (Takagaki and Nanjo, 2013) and procyanidin dimers (Appeldoorn et al., 2009) *in vitro*, and it has been detected in the plasma of healthy volunteers after intake of catechin-rich maritime pine bark extract, (Mülek et al., 2015), indicating its absorption from the large intestine after formation by gut microbiota. Interestingly, the compound was also found to possess much more pronounced *in vitro* anti-inflammatory activity than its precursor catechin (Uhlenhut and Högger, 2012), demonstrating that microbial metabolites of plant constituents can have a strong impact on the pharmacological activity of herbal preparations.

Compound **16** was tentatively assigned to 4-hydroxy-5-(dihydroxyphenyl)valeric acid, that is supposed to be formed in a similar way as **28**, but without water elimination after A- and C-ring cleavage (Monagas et al., 2010; Takagaki and Nanjo, 2013; Figure 6).

The putative next step is oxidation of **28** to 4-oxo-5-(dihydroxyphenyl)valeric acid (Monagas et al., 2010; Takagaki and Nanjo, 2013). Indeed, we detected two metabolites with m/z 223.0604 (**13** and **20**). Based on its MS/MS fragmentation pattern, compound **20** was tentatively assigned to 4-oxo-5-(dihydroxyphenyl)valeric acid (Takagaki and Nanjo, 2013). The compound was detectable only in WBE 2 mg/ml, where it significantly increased over time, but obviously constituted only a minor metabolite. The isomeric compound **13** showed a partly different MS/MS fragmentation pattern. This compound was putatively assigned to 5-(3′,4′,5′-trihydroxyphenyl)-y-valerolactone, a metabolite that might arise from degradation of (epi)gallocatechin (Takagaki and Nanjo, 2010), which is also present at low amounts in WBE (**7**). **13** was only detectable as an intermediate in WBE 2 mg/ml treated with HFS. Since no MS/MS fragmentation for 5-(3′,4′,5′-trihydroxyphenyl)- γ-valerolactone is available in the literature, the assignment of **13** has to be regarded as uncertain.

The major compound **42** and the minor compound **35** shared the same monoisotopic mass (m/z 209.0809), however, their MS/MS fragmentation patterns slightly differed: Referring to Tagakaki et al. (Takagaki and Nanjo, 2013), the major peak eluting at 19.65 min (**42**) was tentatively assigned as 5-(dihydroxyphenyl)valeric acid, while the minor peak (RT 16.61 min) (**35)** was tentatively assigned as 4-hydroxy-5(-hydroxyphenyl)valeric acid. The two compounds have been described as metabolites of (+)-catechin (Takagaki and Nanjo, 2013) and procyanidins (Appeldoorn et al., 2009) *in vitro*. Compound **42** is supposed to be formed by reduction of 4-oxo-5-(dihydroxyphenyl)valeric acid (**20**) (Figure 6). Dehydroxylation of **42**, as well as oxidation and subsequent reduction of the OH group in position 4 of **35** are supposed to result in 5-(3′-hydroxyphenyl)valeric acid (Monagas et al., 2010; Takagaki and Nanjo, 2013). Indeed, the minor metabolite **54** could be tentatively assigned to hydroxyphenylvaleric acid.

Another potential degradation product of **42** is dihydroxyphenylpropionic acid. Also this compound (**17**) was detected as a metabolite in WBE samples incubated with HFS in our study.

Dehydroxylation of **17** as well as β-oxidation of **54** are supposed to lead to the formation of hydroxyphenylpropionic acid (Figure 6). Since the microbial dehydroxylation in ring C of catechin metabolites seems to preferentially occur in position 4′(Monagas et al., 2010; Takagaki and Nanjo, 2013), one would expect 3′-hydroxyphenylpropionic acid as catechin metabolite. Indeed, one of the major metabolites formed in HFS-incubated WBE samples was 3-(3′-hydroxyphenyl)propionic acid (**34**). **34** can be further dehydroxylated to phenylpropionic acid (**49**) that was indeed found to increase over time in HFS- incubated WBE 2 mg/ml; however, it has to be considered that this compound can also arise from naringenin metabolism (Figures 5, 6).

An alternative degradation pathway of **17** leads to the formation of protocatechuic acid (Monagas et al., 2010; Figure 6). Protocatechuic acid (**6**) was present at low amounts in unmetabolized WBE and found to significantly increase upon incubation of both WBE concentrations with HFS.

Further putative degradation products potentially arising from catechin and also naringenin metabolism, like phenylacetic acid, hydroxybenzoic acid and benzoic acid, were not detectable in our samples. However, from experiments with authentic reference compounds we know that these compounds show very weak ionization behavior under the applied experimental conditions.

### Limitations of the study

We found that the combination of microbiome analysis and LC-MS metabolomics applied in this *in vitro* study is a comparably easy and straightforward approach allowing to establish the first idea on interactions taking place between gut microbiota and herbal constituents, and to detect potentially interesting metabolites arising therefrom. However, several limitations have to be considered for this experimental setup:

First, in the applied *in vitro* model, the herbal extract is directly added to HFS; thereby bypassing metabolism and absorption processes possibly taking place in the upper intestinal tract *in vivo*. It has to be taken into consideration that *in vivo*, not all of the extract's constituents might reach the colon, making some of the observed metabolic reactions elusive, as already discussed for the salicylic alcohol derivatives. Therefore, we regard the rather simple experimental setup used in this study only as a first step, allowing the visualization of potentially interesting herb-microbe interactions, but not always correctly representing the *in vivo* situation. For validation of findings, it will be necessary either to mimic digestion and resorption processes by using *in vitro* digestion models, and/ or to perform *in vivo* studies in experimental animals or humans.

Second, it has to be taken into account that the present study has been performed with fecal suspension from a single healthy donor. It is known that the composition of human gut microbial community varies between individuals. Therefore, in order to confirm such results on a broader basis, larger studies with fecal samples from different donors will be necessary.

Third, the applied methods have some limitations: on the one hand, the applied ESI-MS conditions did not allow the satisfactory detection of all analytes present in the samples; in particular, small aromatic compounds like saligenin, 4-hydroxyphenylpropionic acid etc. were found to ionize only weakly, thereby hampering the detection of this group of common microbial metabolites. Application of a second analytical technique, such as GC-MS, might be a solution for future studies. Moreover it has to be considered that MS analyses generally do not allow a full structural assignment unless authentic reference compounds are available.

Fourth, the applied microbiome analysis techniques do not allow a real link between compound turnover and microbial activity. This might be established by using metatranscriptomics as an additional experimental platform.

## Conclusions and outlook

The combined application of 16S rRNA sequencing and LC-MS metabolomics methods in a relatively simple *in vitro* experimental setup allowed first insights not only in the metabolism of WBE constituents by fecal microbiota, but also in the impact of these constituents on fecal microbiota.

Next generation sequencing analysis of microbial DNA revealed that incubation of fecal microbiota with WBE had a significant impact on composition and function of the fecal microbial community. In particular, microorganisms belonging to the genus *Bacteroides* that are known to be involved in the degradation of complex carbohydrates and glycosides obviously profited from WBE addition and hence significantly increased in the fecal sample under study. In order to answer the question whether the observed effects are physiologically relevant, it would be necessary to extend the study to a higher number of fecal samples, and eventually, to perform *in vivo* studies.

Also numerous microbial functions were significantly altered upon WBE addition. However, further metatranscriptomics-based experiments, as well as more targeted investigations e.g., stable isotope probing, would be necessary in order to fully understand the relevance of these findings.

UHPLC-HR-MS analysis of WBE allowed a comprehensive characterization not only of its main constituents, but also of minor compounds, some of which were even unknown so far in *Salix* sp.

Analysis of WBE samples incubated with HFS *in vitro* revealed that most of its constituents markedly decreased over time, indicating that they were readily metabolized by fecal microbiota. Interestingly, for some compound types, the degree of metabolization was lower at the higher WBE concentration, indicating that too high amounts of certain compounds or metabolites thereof might have a negative impact on microbial activity. This aspect should particularly be considered in *in vitro* metabolization studies where often high concentrations of single compounds are incubated with microorganisms.

UHPLC-HRMS analysis also allowed the annotation of numerous microbial metabolites. The fact that we did not work with single compounds but with a total extract containing a high number of constituents made interpretation of these results a complex task. Nevertheless, by comparison of annotated metabolites with literature data, we were able to nicely reconstruct large parts of flavane-3-ol metabolism, and to suggest a potential course for the metabolism of salicylic alcohol derivatives taking place in our *in vitro* experimental setup. The metabolism of naringenin glycosides was more problematic to follow since we were unable to detect 3-(4-hydroxyphenyl)propionic acid, a key metabolite of naringenin metabolism according to the literature, in our samples.

All in all, the obviously efficient metabolization of WBE constituents by gut microbiota should be considered when seeking to unravel the active principles and mechanism of action of willow bark preparations. Since it is known that salicylic alcohol derivatives alone do not account for the anti-inflammatory activity of willow bark, microbial metabolites derived from other compounds like flavonoids and flavan-3-ols, constituents that are supposed to reach the distal gut, should be considered as possible bioactive metabolites of willow bark preparations.

Although the approach we applied has its limitations, it might serve as a tool to provide first ideas on interaction between traditionally used herbal medicines and gut microbiota. Provided that such observations can eventually be confirmed in more detailed *in vitro* studies and *in vivo*, studying interactions between herbal drugs and gut bacteria can be an interesting new possibility to unravel the bioactive principles of herbal medicines.

## Author contributions

E-MP-W performed UHPLC-HRMS analyses, data processing and interpretation, contributed to the incubation experiments, and was a major contributor in writing the manuscript. KK performed microbiome analyses and data processing and contributed in writing the manuscript. CM-E supported the study design, performed the incubation experiments, processed the data and interpreted the microbiome data and was a major contributor in writing the manuscript. RB was the PI, designed and planned the study and contributed to writing and critically revising the manuscript. All authors read and approved the final version of the manuscript and declare to be accountable for all aspects of the work in ensuring that questions related to the accuracy or integrity of any part of the work are appropriately investigated and resolved.

### Conflict of interest statement

The authors declare that the research was conducted in the absence of any commercial or financial relationships that could be construed as a potential conflict of interest.

## Footnotes

^1^http://www.ema.europa.eu/docs/en_GB/document_library/Herbal_-_Herbal_monograph/2017/07/WC500230920.pdf

^2^*The Metabolomics Standards Initiative (MSI) and Core Information for Metabolomics Reporting (CIMR) [Internet]*. [cited 2017 Feb 8]. Available online at: http://cosmos-fp7.eu/msi).
