# Supplementary material for: A Combined LC-MS Metabolomics- and 16S rRNA Sequencing Platform to Assess Interactions between Herbal Medicinal Products and Human Gut Bacteria in Vitro: a Pilot Study on Willow Bark Extract
Source: Front Pharmacol. 2017 Dec 13;8:893. doi: 10.3389/fphar.2017.00893 (PMC5733343; doi:10.3389/fphar.2017.00893)
Supplement: Supplementary file 5 [file DataSheet1.pdf]

Supplementary Data Sheet 1

A combined LC-MS metabolomics- and 16S rRNA sequencing platform to assess interactions between herbal medicinal products and human gut bacteria *in vitro*: A pilot study on willow bark extract

Eva-Maria Pferschy-Wenzig[1], Kaisa Koskinen[2,3], Christine Moissl-Eichinger[*2,3], Rudolf Bauer[*1,3]

[1]Institute of Pharmaceutical Sciences, Department of Pharmacognosy, Universtity of Graz, Universitaetsplatz 4, 8010 Graz, Austria

[2] Section of Infectious Diseases and Tropical Medicine, Department for Internal Medicine, Medical University of Graz, Auenbruggerplatz 15, 8036 Graz, Austria

[3] BioTechMed Graz, Krenngasse 37, 8010 Graz, Austria

*** Correspondence:**

Christine Moissl-Eichinger: christine.moissl-eichinger@medunigraz.at
Rudolf Bauer: rudolf.bauer@uni-graz.at

Supplementary Data

1 Table S6: Details on identification of compounds significantly changing over incubation time

#	RT (min)	measured m/z [ion species]	indicative MS/MS fragments (relative intensity)	Monoisotopic mass	Molecular formula	Δ (ppm)	Identification<br><br>(+InchI key for unambiguously identified compounds)	Literature/Database
1	1.33	377.0862 $[M+Cl]^-$	377 (60) 341 (100) 215 (20) 179 (25) 161 (5) 139 (5) 119 (10) 89 (20)	342.1166	$C_{12}H_{22}O_{11}$	4.14	saccharose[b]	http://www.hmdb.ca/
2	1.43	133.013 $[M-H]^-$	133 (40) 115 (100) 97 (10) 89 (10) 73 (10) 71 (45)	134.0203	$C_4H_6O_5$	-1.35	malic acid[b]	https://metlin.scripps.edu/ [b]
3	1.63	191.019 $[M-H]^-$	191 (30) 173 (5) 129 (10) 111 (100) 87 (35) 85	192.0263	$C_6H_8O_7$	1.94	citric acid[a]<br><br>KRKNYBCHXYNGOX-UHFFFAOYSA-N	

			(25)					
4	1.96	117.018 [M-H]$^-$	117 (20) 99 (10) 73 (100)	118.0252	$C_4H_6O_4$	-2.42	succinic acid[a]<br><br>KDYFGRWQOYBRFD-UHFFFAOYSA-N	
5	2.65	157.0492 [M-H]$^-$	157 (100) 139 (2) 113 (5) 95 (100)	158.0565	$C_7H_{10}O_4$	-2.00	isopropylmaleate[b]	http://www.hmdb.ca/
6	4.93	153.0179 [M-H]$^-$	153 (35) 109 (100)	154.0252	$C_7H_6O_4$	-2.32	protocatechuic acid[a,+]<br><br>YQUVCSBJEUQKSH-UHFFFAOYSA-N	(1)[+]
7	5.17	305.067 [M-H]$^-$	305 (50) 261 (10) 221 (10) 219 (15) 179 (20) 175 (100) 167 (15) 165 (20) 147 (35) 139 (15) 137 (15) 125 (60)	306.0742	$C_{15}H_{14}O_7$	5.12	(epi)gallocatechin[b,#]	https://metlin.scripps.edu/ [b]; (2)[#]

8	5.63	109.028 [M-H]$^-$	109 (100) 91 (3) 81 (3)	110.0352	$C_6H_6O_2$	-2.99	catechol[b]	https://metlin.scripps.edu/ [b]
9	6.08	329.0882 [M-H]-	175 (15) 169 (15) 167 (100) 152 (10) 123 (15) 108 (10)	330.0957	$C_{14}H_{18}O9$	5.26	vanillic acid hexoside[b,#]	(3)[b], (4)[#]
10	6.72	321.075 [M+^{35}Cl]$^-$ (35); 323.072 [M+^{37}Cl]$^-$ (12); 331.104 [M-H+HCOOH]$^-$ (100)	123 (100)	286.1055	$C_{13}H_{18}O_7$	3.78	salicin[a, #] NGFMICBWJRZIBI-UJPOAAIJSA-N	(5)[#], (6)[#]
11	6.98	226.0539 [M-H]$^-$	199 (7) 181 (100) 159(7) 153 (20) 139 (5) 131 (5)	227.0611	$C_{10}H_{13}O_3NS$	1.94	saligenin-cysteine adduct[b]	

			120 (85)					
12	7.06	123.0439 [M-H]$^-$	123 (30)<br>121 (100)<br>93 (30)	124.0512	$C_7H_8O_2$	-1.51	saligenin[a,#]<br><br>CQRYARSYNCAZFO-UHFFFAOYSA-N	(5)[#]
13	7.68	223.0604 [M-H]-, 259.0375 [M+^{35}Cl]-	223 (100)<br>179 (50)<br>138 (10)	224.0677	$C_{11}H_{12}O_5$	1.26	5-(3´,4´,5´-trihydroxyphenyl)-y-valerolactone[b,+]	(7)[+]
14	7.70	153.0179 [M-H]$^-$	153 (60)<br>109 (100)<br>108 (25)	154.0252	$C_7H_6O_4$	-2.25	gentisic acid[a]<br><br>WXTMDXOMEHJXQO-UHFFFAOYSA-N	
15	7.72	137.023 [M-H]$^-$	137 (20)<br>93 (100)	138.0301	$C_7H_6O_3$	-3.51	4-hydroxybenzoic acid[a,+]<br><br>FJKROLUGYXJWQN-UHFFFAOYSA-N	
16	8.88	225.076 [M-H]$^-$	225 (30)<br>207 (15)<br>181 (10)<br>163 (55)<br>123 (100)<br>101 (80)	226.0834	$C_{11}H_{14}O_5$	1.6	4-hydroxy-5-(3,4-dihydroxyphenyl)valeric acid[b,+]	(8)[b,+]

17	9.13	181.049 [M-H]-	181 (80) 153 (85) 137 (100) 121 (20) 109 (20) 93 (5) 61 (7) 59 (45)	182.0570	$C_9H_{10}O_4$	2.07	dihydrocaffeic acid[a,+] DZAUWHJDUNRCTF-UHFFFAOYSA-N	(8)[+], (9)[+]
18	9.23	577.1357 [M-H]-	577 (7) 451 (15) 425 (25) 407 (85) 289 (100) 245 (17) 179 (5) 161 (25) 151 (5) 137 (10) 125 (85)	578.143	$C_{30}H_{26}O_{12}$	2.82	epi)catechin-(epi)catechin[b,#]	https://metlin.scripps.edu/[b], (2)[#]
19	9.62	289.072 0[M-H]-	289 (100) 245 (35) 221 (10) 205 (20) 203 (25) 179 (17) 175 (15) 151 (15) 137 (15) 125 (27)	290.0793	$C_{15}H_{14}O_6$	4.59	catechin[a,#] PFTAWBLQPZVEMU-DZGCQCFKSA-N	(2)[#]

20	9.79	223.0604 [M-H]$^-$	223 (70)<br>205 (5)<br>195 (10)<br>179 (30)<br>161 (10)<br>123 (100)	224.0677	$C_{11}H_{12}O_5$	1.44	4-oxo-5-(dihydroxyphenylvaleric)acid[b,+]	(8)[b,+]
21	9.87	153.0179 [M-H]$^-$	153 (30)<br>109 (100)	154.0251	$C_7H_6O_4$	-2.37	dihydroxybenzoic acid[b]	https://metlin.scripps.edu/[b]
22	10.52	131.0698 [M-H]$^-$	131 (90)<br>119 (35)<br>85 (100)<br>62 (5)	132.0771	$C_6H_{12}O_3$	-3.74	hydroxy(iso)caproic acid [b, +]	http://www.hmdb.ca[b]; (10)[+]
23	10.54	363.085 [M+^{35}Cl]$^-$, 365.083 [M+^{37}Cl]$^-$, 373.1143 [M-H+HCOOH]$^-$	175 (30)<br>123 (100)<br>59 (25)	328.1161	$C_{15}H_{20}O_8$	3.82	acetylsalicin[b,#]	(5)[b,#], (11)[#]
24	10.88	131.0698 [M-H]$^-$	131 (90)<br>119 (20)<br>85 (100)	132.0771	$C_6H_{12}O_3$	-3.68	hydroxy(iso)caproic acid[b, +]	http://www.hmdb.ca[b]; (10)[+]
25	10.92	353.0882	191 (100)	354.0955	$C_{16}H_{18}O_9$	4.48	chlorogenic acid[a,#]	(12)[#]

		[M-H]$^-$					CWVRJTMFETXNAD-JUHZACGLSA-N	
26	10.95	865.1994 [M-H]$^-$	739 (3)<br>713 (5)<br>577 (15)<br>543 (7)<br>525 (5)<br>451 (15)<br>449 (15)<br>425 (20)<br>407 (55)<br>289 (50)<br>287 (45)<br>261 (7)<br>245 (15)<br>243 (25)<br>161 (30)<br>125 (100)	866.2067	$C_{45}H_{38}O_{18}$	2.47	(epi)catechin-(epi)catechin-(epi)catechin[b,#]	(13), (14)[b]; (2), (12)[#]
27	11.38	407.1120 [M-H+^{35}Cl]$^-$; 417.1408 [M-H+HCOOH]$^-$	209 (100)<br>194 (10)<br>175 (50)<br>145 (10)	372.1421	$C_{17}H_{24}O_9$	4.44	syringin[b,#]	(15)[b], (6)[#]
28	12.34	163.0751 [M-H-CO_2]$^-$, 207.0654 [M-H]$^-$,	207 (100)<br>163 (75)<br>122 (20)	208.0726	$C_{11}H_{12}O_4$	0.07	5-(dihydroxyphenyl)-y-valerolactone[b,+]	(8), (1)[b,+]

		243.0425 [M+^{35}Cl]$^-$, 245.04396 [M+^{37}Cl]$^-$						
29	12.40	447.1514 [M-H+HCOOH]$^-$	401 (100) 293 (3) 269 (45) 233 (5) 161 (30) 143 (3) 113 (10) 101 (20) 89 (5) 71 (15)	402.1539	$C_{18}H_{26}O_{10}$	4.50	benzyl-hexoside-pentoside[b]	(16)[b]
30	12.42	319.0462 [M-H]$^-$	319 (5) 301 (15) 257 (15) 215(15) 193 (100) 179 (20) 175 (25) 167 (10) 151 (10) 125 (40)	320.0534	$C_{15}H_{12}O_8$	4.16	ampelopsin[b,#]	(17)[b], (6)[#]
31	12.86	383.0083 [M-H]$^-$	383 (20) 303 (100) 285 (80)	384.01626	$C_{15}H_{12}O_{10}S$	1.56	diyhdroquercetin sulfate[b]	(18)[b]

			275 (10)<br>259 (5)<br>241 (3)<br>217 (3)<br>177 (25)<br>153 (3)<br>125 (40)					
32	12.89	447.1513 [M-H+HCOOH]$^-$	401 (50)<br>269 (100)<br>175 (5)<br>161 (35)<br>113 (15)<br>101 (20)<br>89 (3) 85 (7) 71 (17)	402.1541	$C_{18}H_{26}O_{10}$	4.95	benzyl-hexoside-pentoside[b]	(16)[b]
33	14.39	363.0854 [M-H+^{35}Cl]$^-$; 365.0825 [M-H+^{37}Cl]$^-$; 373.1141 [M-H+HCOOH]$^-$	175 (3)<br>123 (100)<br>59 (33)	328.1159	$C_{15}H_{20}O_8$	3.42	acetylsalicin[b,#]	(5)[b,#]
34	14.65	165.0543 [M-H]$^-$, 121.0643 [M-H-CO_2]$^-$, 119.0486	136 (5)<br>121 (100)<br>119 (45)	166.0616	$C_9H_{10}O_3$	-1.88	3-(3-hydroxyphenyl)propionic acid[a, +]<br><br>QVWAEZJXDYOKEH-UHFFFAOYSA-N	(8)[a,+]

		[M-H-HCOOH]$^-$						
35	16.61	209.081 [M-H]$^-$	209 (100)<br>191 (35)<br>165 (25)<br>151 (15)<br>147 (10)<br>123 (20)	210.0882	$C_{11}H_{14}O_4$	0.45	4-hydroxy-5-(hydroxyphenyl))valeric acid [b,+]	(8)[b,+]
36	17.78	303.0514<br><br>[M-H]$^-$	303 (15)<br>285 (85)<br>275 (15)<br>259 (15)<br>217 (15)<br>179 (10)<br>177 (25)<br>175 (30)<br>125 (100)	304.0585	$C_{15}H_{12}O_7$	4.62	dihydroquercetin[a, +]<br><br>CXQWRCVTCMQVQX-LSDHHAIUSA-N	(6)[#]
37	17.89	433.1143 [M-H]$^-$	433 (15)<br>271 (100)<br>313 (5)<br>151 (25)<br>119 (5)	434.1215	$C_{21}H_{22}O_{10}$	3.64	naringenin hexoside[b,#]	(5)[#]
38	17.99	137.0229 [M-H]$^-$	93 (100)	138.0302	$C_7H_6O_3$	-3.29	salicylic acid[a]<br><br>YGSDEFSMJLZEOE-UHFFFAOYSA-N	(5)[#]

39	18.34	473.1665 [M-H+HCOOH]$^-$	427 (15) 293 (40) 233 (7) 161 (85) 149 (30) 143 (10) 131 (30) 125 (40) 113 (35) 101 (45) 89 (100) 85 (15) 73 (10) 71 (90) 59 (45)	428.1683	$C_{20}H_{28}O_{10}$	3.08	rosarin[b]	(19)[b]
40	18.49	459.107 [M+^{35}Cl]$^-$; 461.1031 [M+^{37}Cl]$^-$; **469.1353** [M-H+HCOOH]$^-$; 486.1256	423 (30) 317 (5) 161 (40) 155 (100) 137 (35) 123 (80) 111 (75) 101 (10) 83 (30) 71 (15)	424.1371	$C_{20}H_{24}O_{10}$	1.79	salicortin [b,#]	(5)[b,#]
41	18.84	433.1143	271 (100) 313 (5)	434.1215	$C_{21}H_{22}O_{10}$	3.09	naringenin-hexoside ((-)-	(5)[b,#]

		[M-H]$^-$	151 (25)				naringenin-5-glucoside?) [b,#]	
42	19.65	209.0809 [M-H]$^-$, 245.058 [M+Cl]-, 293.0341	209 (100) 191 (10) 165 (3) 147 (3) 135 (7) 121 (3) 109 (3)	210.0882	$C_{11}H_{14}O_4$	0.31	5-(dihydroxyphenyl)valeric acid[b,+]	(8)[b,+]
43	19.66	473.1664 [M-H+HCOOH]$^-$	427 (20) 293 (70) 233 (15) 191 (20) 161 (65) 149 (40) 143 (10) 131 (25) 125 (40) 113 (30) 101 (40) 89 (100) 71 (85) 59 (45)	428.1690	$C_{20}H_{28}O_{10}$	2.94	cinnamyl-(6`-O-xylopyranosyl)-O-glucopyranoside[b]	(19)[b]
44	20.70	463.0884 [M-H]$^-$	463 (55) 301 (70) 300 (100) 271 (5)	464.0957	$C_{21}H_{20}O_{12}$	4.27	hyperoside[a,#]<br><br>OVSQVDMCBVZWGM-DTGCRPNFSA-N	(20)[#]

			255 (3) 179 (3) 151 (3)					
45	20.95	487.1827 [M-H+HCOOH]$^{-}$	441 (15) 340 (3) 307 (50) 247 (30) 205 (5) 175 (20) 163 (55) 145 (15) 125 (75) 119 (45) 113 (7) 103 (70) 101 (90) 97 (15) 89 (60) 85 (20) 81 (10) 73 (40) 71 (57) 59 (100)	442.1840	$C_{21}H_{30}O_{10}$	2.80	cinnamylhexoside-deoxyhexoside [b]	https://metlin.scripps.edu/[b]
46	21.52	489.1983 [M-H+HCOOH]$^{-}$	443 (95) 297 (100) 161 (25) 143 (3) 113 (10) 101 (30) 89 (5) 83	444.2001	$C_{21}H_{32}O_{10}$	3.42	dihydrocinnamylhexoside-deoxyhexoside[b]	(21)[b]

			(7) 81 (10) 73 (15) 71 (20)					
47	21.90	433.1149 $[M-H]^-$	271 (100) 175 (5) 151 (15)119 (5)	434.1215	$C_{21}H_{22}O_{10}$	1.58	naringenin-7-glucoside[a,#]<br><br>DLIKSSGEMUFQOK-SFTVRKLSSA-N	(5)[#]
48	22.34	441.0963 $[M+^{35}Cl]^-$; 451.125 $[M-H+HCOOH]^-$	283 (60); 163 (7); 121 (100)	406.1268	$C_{20}H_{22}O_9$	3.28	salireposide [b,#]	(5)[b,#]
49	24.15	149.0593 $[M-H]^-$	149 (100) 121 (50) 105 (5) 93 (10)	150.067	$C_9H_{10}O_2$	-0.35	phenylpropionic acid[b,+]	http://www.hmdb.ca[b]; (1),'(22), (23)[+]
50	24.79	501.1169 $[M+^{35}Cl]^-$; 502.1203; 503.1141 $[M+^{37}Cl]^-$; 511.1457 $[M-H+HCOOH]^-$; 528.1355	465 (50) 423 (5) 405 (20) 299 (2) 271 (2) 155 (100) 137 (70) 123 (85) 111 (75) 109 (25)	466.1475	$C_{22}H_{26}O_{11}$	1.86	acetylsalicortin [b,#]	(5)[b,#]

			83 (60)<br>59 (40					
51	24.97	423.1659 [M-H]-	163 (20)<br>145 (100)	424.173	$C_{21}H_{28}O_9$	4.23	grandidentatin/isograndidentatin [b,#]	(24)[b], (11)[#]
52	25.40	433.1138 [M-H]-	433 (5)<br>313 (5)<br>271 (100)<br>151 (30)<br>119 (5)	434.1211	$C_{21}H_{22}O_{10}$	3.99	isosalipurposide[b,#]	(5)[b,#]
53	25.46	423.1666 [M-H]-	163 (20);<br>145 (100)	424.174	$C_{21}H_{28}O_9$	4.02	grandidentatin/isograndidentatin [b,#]	(24)[b], (11)[#]
54	25.87	193.0859 [M-H]-	193 (100)<br>175 (25)<br>149 (10)	194.093	$C_{11}H_{14}O_3$	0.00	hydroxyphenylvaleric acid[b,+]	(8)[b,+]
55	27.51	435.1299 [M-H+HCOOH]-	389 (100)<br>283 (15)<br>123 (5)	390.132	$C_{20}H_{24}O_9$	3.07	benzoylsalicin [b,#]	(5)[b,#]

56	30.77	271.061 [M-H]$^-$	271 (100) 177 (10) 151 (60) 119 (20) 107 (7) 93 (7)	272.0683	$C_{15}H_{12}O_5$	4.58	naringenin[a,#]<br><br>FTVWIRXFELQLPI-UHFFFAOYSA-N	(25)[#]
57	32.46	649.1785 [M-H+HCOOH]$^-$	603 (10) 543 (5) 465 (20) 447 (10) 405 (10)<br><br>341 (3) 155 (40) 137 (45) 123 (47) 111 (100) 109 (45) 93 (10) 83 (35) 59 (20)	604.1893	$C_{29}H_{32}O_{14}$	2.45	HCH-acetylsalicortin[b,#]	(26)[b], (27)[#]
58	35.54	563.1329 [M+^{35}Cl]-;<br><br>565.1292 M+^{37}Cl]-<br><br>573.1616 [M-H+HCOOH	527 (7) 405 (15) 155 (30) 137 (25) 123 (10) 121 (100) 11 (20) 109 (10)	528.163	$C_{27}H_{28}O_{11}$	1.73	tremulacin [b,#]	(5)[b,#]

		]⁻; 590.1515	83 (17)					

a: identified by comparison with authentic reference compound; b: tentative identification based on monoisotopic mass and comparison with fragmentation patterns in literature or databases (HMDB, Metlin, Massbank, mzCloud); structural isomers cannot be ruled out

# described in the literature in Salix sp.

+ described in the literature as intestinal metabolite

2 Identification details for selected compounds:

Compound **11**: The compound was tentatively assigned as salicin-cysteine adduct, based on its molecular formula and MS/MS fragmentation pattern. Since cysteine adducts are no common microbial metabolites, we performed the following control experiment in order to find out how the observed adduct is formed: pure saligenin was mixed with cysteine (1 mg/ml each) in PBS buffer containing no human fecal suspension. In order to test whether the adduct is formed during analysis in the ESI source, one sample was measured directly after mixing saligenin and cysteine, however, no saligenin-cysteine adduct was detectable. Therefore, two further samples were prepared in the same way and incubated for 24 h, one at room temperature, and one at 37°C, mimicking the experimental conditions of HFS incubation. Indeed, after incubation, the adduct was detectable, with trace levels in the sample incubated at RT and high levels in the sample incubated at 37°C, indicating that the compound is an artefact which is spontaneously formed under the applied experimental conditions (Figure S1).

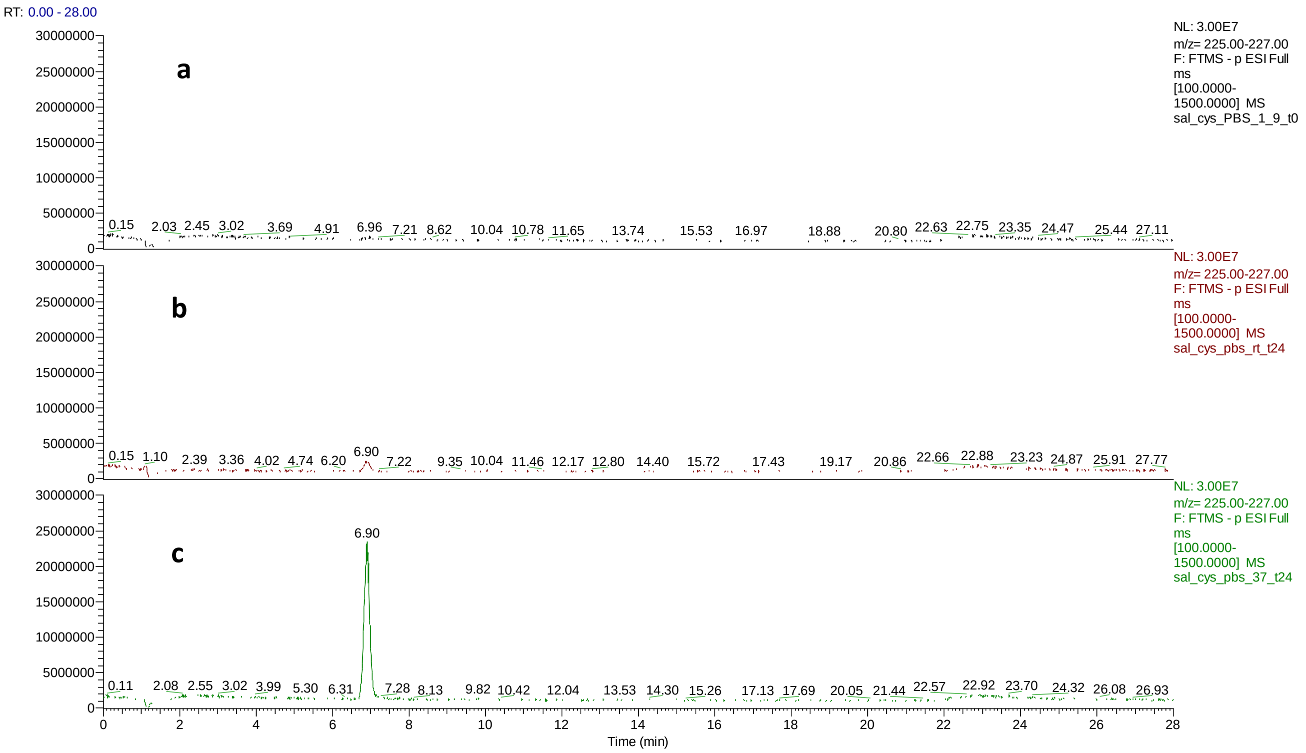

Figure S1: ESI negative mode extracted ion chromatograms (m/z 225-227) of saligenin-cysteine mixture in PBS buffer **(a)** directly after mixing saligenin with cysteine **(b)** after 24 h of incubation at room temperature **(c)** after 24 h of incubation at 37°C.

<u>Compounds **23** and **33**</u> were tentatively identified as acetylsalicins based on their molecular formula and on their MS/MS fragmentation patterns that were similar to the ones described for fragilin (6´-acetylsalicin) by Kammerer et al(5). The occurrence of fragilin has been described in pharmaceutical willow bark preparations; therefore, we assume that one of the two detected isomers is fragilin. The second isomer might bear the acetyl group in a different position. For example , 2´- and 3´-O-acetylsalicin have been isolated from *S. pseudolasiogyne* (11).

<u>Compounds **27**, **31**, **35** and **40**</u> all possess the same HR mass and molecular formula, namely $C_{21}H_{22}O_{10}$.There are several structural isomers possessing this molecular formula described in *Salix* sp.: the flavonone glycosides (+)- and (-)-naringenin-5-glucoside (salipurposide) and naringenin-7-

glucoside, as well as the chalcone glucoside isosalipurposide. Since these compounds share the same MS/MS fragmentation pattern, they cannot be discriminated on this basis. However, the chalcone isosalipurposide has a distinct UV spectrum (λ_{max} 370 nm vs. λ_{max} 280 nm for naringenin glycosides).

When we analyzed authentic naringenin-7-glucoside, the compound could be assigned to peak **47**. Low levels of **47** were detectable at t0 in WBE 10 mg/ml. In WBE 2 mg/ml, the levels of **47** were that low that it was not included in the processed LCMS data. However, inspection of the raw data showed that trace amounts of compound **47** were present in WBE 2 mg/ml at t0 as well. Concerning the two major naringenin hexoside peaks, we tentatively assigned compound **37** to (+)-naringenin-5-glucoside and compound **41** to its diastereomer (-)-naringenin-5-glucoside on the basis of their elution order described in earlier studies(5). Peak **52**, eluting at 25.40 min, was tentatively assigned to the chalcone isosalipurposide due to its typical UV spectrum and due to the elution order that was in accordance with the one in the study by Kammerer et al. (5).

<u>Compound 28:</u> The compound´s fragmentation pattern was in accordance with that of 5-(dihydroxyphenyl)valerolactone(8).

<u>Compound 20:</u> Based on its MS/MS fragment m/z 123, that was reported for 4-oxo-5-(dihydroxyphenyl)valeric acid, compound **20** was tentatively assigned to this metabolite(8).

<u>Compounds **42** and **35**</u> shared the same monoisotopic mass (m/z 209.0809), however, their MS/MS fragmentation patterns slightly differed: In its MS/MS spectrum, compound **42** displayed a fragment with m/z 135, which was not present in the MS/MS spectrum of compound **35**. According to Tagakaki et al.(8), this fragment occurs in the MS/MS spectrum 5-(3,4-dihydroxyphenyl)valeric acid, while it does not occur in the MS/MS spectrum of 4-hydroxy-5-(3-hydroxyphenyl)valeric acid. Therefore, the major peak with m/z 209.0809 eluting at 19.65 min (**42**) was tentatively assigned as 5-

(dihydroxyphenyl)valeric acid, while the minor peak (m/z 209.0809, RT 16.61 min) (**35)** was tentatively assigned as 4-hydroxy-5(-hydroxyphenyl)valeric acid.

References:

1. Goodrich KM, Neilson AP. Simultaneous UPLC-MS/MS analysis of native catechins and procyanidins and their microbial metabolites in intestinal contents and tissues of male Wistar Furth inbred rats. J Chromatogr B Anal Technol Biomed Life Sci. Elsevier B.V.; 2014;958:63–74.

2. Jürgenliemk G, Petereit F, Nahrstedt A. Flavan-3-ols and procyanidins from the bark of Salix purpurea L. Pharmazie. 2007;62(3):231–4.

3. Ammar S, Contreras M del M, Belguith-Hadrich O, Bouaziz M, Segura-Carretero A. New insights into the qualitative phenolic profile of Ficus carica L. fruits and leaves from Tunisia using ultra-high-performance liquid chromatography coupled to quadrupole-time-of-flight mass spectrometry and their antioxidant activity. RSC Adv. 2015;5(26):20035–50.

4. Liao Y, Zhou G, Wu G, Pan L, Fu H, Ding J, et al. A new chromone glycoside from leaves of Salix matsudana. Zhongcaoyao. 2014;45(20):2887–9.

5. Kammerer B, Kahlich R, Biegert C, Gleiter CH, Heide L. HPLC-MS/MS analysis of willow bark extracts contained in pharmaceutical preparations. Phytochem Anal. 2005;16(6):470–8.

6. Agnolet S, Wiese S, Verpoorte R, Staerk D. Comprehensive analysis of commercial willow bark extracts by new technology platform: Combined use of metabolomics, high-performance liquid chromatography-solid-phase extraction-nuclear magnetic resonance spectroscopy and high-resolution radical scavengin. J Chromatogr A. Elsevier B.V.; 2012;1262:130–7.

7. Takagaki A, Nanjo F. Metabolism of (-)-epigallocatechin gallate by rat intestinal flora. J Agric Food Chem. 2010;58(2):1313–21.

8. Takagaki A, Nanjo F. Catabolism of (+)-catechin and (-)-epicatechin by rat intestinal microbiota. J Agric Food Chem. 2013;61(20):4927–35.

9. Monagas M, Urpi-Sarda M, Sánchez-Patán F, Llorach R, Garrido I, Gómez-Cordovés C, et al. Insights into the metabolism and microbial biotransformation of dietary flavan-3-ols and the bioactivity of their metabolites. Food Funct. 2010;1(3):233–53.

10. Zheng X, Xie G, Zhao A, Zhao L, Yao C, Chiu NHL, et al. The footprints of gut microbial-mammalian co-metabolism. J Proteome Res. 2011;10(12):5512–22.

11. Yang H, Lee SH, Sung SH, Kim J, Kim YC. Neuroprotective compounds from Salix pseudo-lasiogyne twigs and their anti-amnesic effects on scopolamine-induced memory deficit in mice. Planta Med. 2013;79(1):78–82.

12. Zaiter A, Becker L, Petit J, Zimmer D, Karam M-C, Baudelaire É, et al. Antioxidant and

antiacetylcholinesterase activities of different granulometric classes of Salix alba (L.) bark powders. Powder Technol. Elsevier B.V.; 2016;301:649–56.

13. Piccinelli AL, Pagano I, Esposito T, Mencherini T, Porta A, Petrone AM, et al. HRMS Profile of a Hazelnut Skin Proanthocyanidin-rich Fraction with Antioxidant and Anti-Candida albicans Activities. J Agric Food Chem. 2016;64(3):585–95.

14. Bijttebier S, Van Der Auwera A, Voorspoels S, Noten B, Hermans N, Pieters L, et al. A First Step in the Quest for the Active Constituents in Filipendula ulmaria (Meadowsweet): Comprehensive Phytochemical Identification by Liquid Chromatography Coupled to Quadrupole-Orbitrap Mass Spectrometry. Planta Med. 2016;82(6):559–72.

15. Tótha G, Barabás C, Tóth A, Kéry Á, Béni S, Boldizsár I, et al. Characterization of antioxidant phenolics in Syringa vulgaris L. flowers and fruits by HPLC-DAD-ESI-MS. Biomed Chromatogr. 2016;30(6):923–32.

16. Delgado De La Torre MP, Priego-Capote F, Luque De Castro MD. Tentative identification of polar and mid-polar compounds in extracts from wine lees by liquid chromatography-tandem mass spectrometry in high-resolution mode. J Mass Spectrom. 2015;50(6):826–37.

17. Yang WZ, Ye M, Qiao X, Wang Q, Bo T, Guo DA. Collision-induced dissociation of 40 flavonoid aglycones and differentiation of the common flavonoid subtypes using electrospray ionization ion-trap tandem mass spectrometry and quadrupole time-of-flight mass spectrometry. Eur J Mass Spectrom. 2012;18(6):493–503.

18. Vacek J, Papoušková B, Vrba J, Zatloukalová M, Křen V, Ulrichová J. LC-MS metabolic study on quercetin and taxifolin galloyl esters using human hepatocytes as toxicity and biotransformation in vitro cell model. J Pharm Biomed Anal. 2013;86:135–42.

19. Tolonen A, Hohtola A, Jalonen J. Comparison of electrospray ionization and atmospheric pressure chemical ionization techniques in the analysis of the main constituents from Rhodiola rosea extracts by liquid chromatography/mass spectrometry. J Mass Spectrom. 2003;38(8):845–53.

20. Nybakken L, Julkunen-Tiitto R. Gender differences in Salix myrsinifolia at the pre-reproductive stage are little affected by simulated climatic change. Physiol Plant. 2013;147(4):465–76.

21. Tolonen A, György Z, Jalonen J, Neubauer P, Hohtola A. LC/MS/MS identification of glycosides produced by biotransformation of cinnamyl alcohol in Rhodiola rosea compact callus aggregates. Biomed Chromatogr. 2004;18(8):550–8.

22. Rechner AR, Smith MA, Kuhnle G, Gibson GR, Debnam ES, Srai SKS, et al. Colonic metabolism of dietary polyphenols: Influence of structure on microbial fermentation products. Free Radic Biol Med. 2004;36(2):212–25.

23. Orrego-Lagarón N, Vallverdú-Queralt A, Martínez-Huélamo M, Lamuela-Raventos RM, Escribano-Ferrer E. Metabolic profile of naringenin in the stomach and colon using liquid chromatography/electrospray ionization linear ion trap quadrupole-Orbitrap-mass

spectrometry (LC-ESI-LTQ-Orbitrap-MS) and LC-ESI-MS/MS. J Pharm Biomed Anal. Elsevier B.V.; 2016;120:38–45.

24. Jervis J, Hildreth SB, Sheng X, Beers EP, Brunner AM, Helm RF. A metabolomic assessment of NAC154 transcription factor overexpression in field grown poplar stem wood. Phytochemistry. Elsevier Ltd; 2014;115:112–20.

25. Freischmidt A, Jürgenliemk G, Kraus B, Okpanyi SN, Müller J, Kelber O, et al. Contribution of flavonoids and catechol to the reduction of ICAM-1 expression in endothelial cells by a standardised Willow bark extract. Phytomedicine. Elsevier GmbH.; 2012;19(3–4):245–52.

26. Keefover-Ring K, Ahnlund M, Abreu IN, Jansson S, Moritz T, Albrectsen BR. No evidence of geographical structure of salicinoid chemotypes within Populus tremula. PLoS One. 2014;9(10).

27. Merken HM, Clausen P. Phenolic glycosides from Salix lasiandra. J Nat Prod. 1992;55(7):970–3.
